# The Rotary Zone Thermal Cycler: A Low-Power System Enabling Automated Rapid PCR

**DOI:** 10.1371/journal.pone.0118182

**Published:** 2015-03-31

**Authors:** Michael S. Bartsch, Harrison S. Edwards, Daniel Lee, Caroline E. Moseley, Karen E. Tew, Ronald F. Renzi, James L. Van de Vreugde, Hanyoup Kim, Daniel L. Knight, Anupama Sinha, Steven S. Branda, Kamlesh D. Patel

**Affiliations:** 1 Sandia National Laboratories, Livermore, CA, United States of America; 2 SequoiaTek Corporation, Logan, UT, United States of America; Northeastern University, UNITED STATES

## Abstract

Advances in molecular biology, microfluidics, and laboratory automation continue to expand the accessibility and applicability of these methods beyond the confines of conventional, centralized laboratory facilities and into point of use roles in clinical, military, forensic, and field-deployed applications. As a result, there is a growing need to adapt the unit operations of molecular biology (e.g., aliquoting, centrifuging, mixing, and thermal cycling) to compact, portable, low-power, and automation-ready formats. Here we present one such adaptation, the rotary zone thermal cycler (RZTC), a novel wheel-based device capable of cycling up to four different fixed-temperature blocks into contact with a stationary 4-microliter capillary-bound sample to realize 1-3 second transitions with steady state heater power of less than 10 W. We demonstrate the utility of the RZTC for DNA amplification as part of a highly integrated rotary zone PCR (rzPCR) system that uses low-volume valves and syringe-based fluid handling to automate sample loading and unloading, thermal cycling, and between-run cleaning functionalities in a compact, modular form factor. In addition to characterizing the performance of the RZTC and the efficacy of different online cleaning protocols, we present preliminary results for rapid single-plex PCR, multiplex short tandem repeat (STR) amplification, and second strand cDNA synthesis.

## Introduction

Since its introduction [1], the polymerase chain reaction (PCR) has become all but ubiquitous in molecular biology as a tool for nucleic acid amplification, quantitation, and detection. The isolation and commercialization of an ever expanding collection of fast, thermostable, and inhibitor-tolerant polymerases [2,3] in conjunction with the availability of increasingly robust, affordable, and user-friendly PCR kits [4,5] has dramatically expanded the accessibility and impact of these methods to applications ranging from pathogen detection and clinical diagnostics [6–9] to gene expression analysis [10], forensics [11–13], and food safety [14–16] and authentication [17,18]. At the same time, progress in automated sample processing driven by advances in microfluidic and lab-on-chip techniques [19–22] is making the use of PCR-enabled approaches outside the controlled confines of the molecular biology laboratory increasingly practicable [23–25].

Despite these trends and the continuing prevalence of PCR methods, the fundamental challenges of utilizing PCR in dispersed applications (i.e., portable, deployable, point-of-care, low-resource) remain significant. Emerging cycle-free alternatives [26–28] such as isothermal amplification seek to address the primary perceived weaknesses of extra-laboratory PCR, namely speed, logistics, and ease of use. A system that effectively addresses these three criteria could substantially enable the use of proven PCR methods in an on-demand fashion for time-sensitive, decentralized applications including point-of-care clinical diagnostics [29–31], kinship verification at border crossings or refugee facilities [32], persistently deployed aerosol sampling [33–35] and biowarfare agent detection [36–39], genotyping for military expeditionary [40,41] and criminal investigative forensics [42,43], post-disaster identification of remains [44,45], and pathogen detection for global health and infectious disease surveillance [46–48].

An enormous body of research [49] has focused on developing faster thermal cycling technologies for PCR, inspired in part by the observation that much of the canonical 20–40 cycle denature (90–98°C), anneal (50–65°C), and elongation (70–80°C) temperature sequence can be abbreviated without sacrificing performance [50]. In extra-laboratory applications, the most significant speed benefit may be realized simply by performing PCR and associated operations locally rather than transporting samples to a central lab facility. Critical logistical factors affecting the suitability of dispersed PCR include power requirements and the need to replace or resupply consumables, but size and weight (i.e., portability), environmental compatibility, ruggedization, and PCR reagent stability [4] must all be considered. Lastly, the potential single-molecule sensitivity of PCR requires even expert technicians in controlled laboratory environments to employ careful molecular biology hygiene [51,52], a level of rigor that must be replicated through containment, integration, and automation to enable non-experts to operate PCR-enabled systems outside the laboratory.

To address the three critical elements of extra-laboratory PCR—speed, logistics, and operability—while facilitating modular integration with other functional units, this article introduces a new concept for rapid cycling, the rotary zone thermal cycler (RZTC). Described in section 1.3 below, the unique design of the RZTC enables rapid temperature transitions to reduce cycle time while minimizing power consumption. Built around an RZTC, the rotary zone PCR (rzPCR) module addresses the requirement for simplified logistics and ease of use by providing an integrated, self-contained, and user-friendly system offering robust operation and highly automated sample loading, unloading, amplification, and between-run cleaning capabilities.

## Background: Thermal Cycling Technologies

While a comprehensive survey of DNA amplification systems and approaches is beyond the scope of this article, numerous reviews of this prolific field have been published over the years with particular emphasis on microfluidic and chip-based PCR [21,25,49,53–57]. As these reviews illustrate, most thermal cyclers can be classified as either temporal or spatial in nature [58]. In temporal cyclers, the sample remains stationary while the temperature changes, whereas in spatial cyclers the sample moves among different fixed-temperature zones to yield the desired temperature history. Here we discuss a representative sampling of thermal cycler designs with an emphasis on those that are amenable to integration and those that have been successfully applied in integrated or automated systems with potential for extra-laboratory operation. To provide a basis for comparison, we begin with a brief survey of commercial PCR instruments.

### 1.1. Commercial Bench-top PCR

Most commercial PCR systems are temporal cycling designs that rely on Peltier thermoelectric (TE) heater/cooler elements to modulate the temperature of a sample-bearing thermal block, a power-intensive approach (typically 200–1000 W) primarily suited to operation in high-resource laboratories. Typified by instruments like the Agilent SureCycler 8800, Bio-Rad C1000, Eppendorf Mastercycler Pro, Life Technologies GeneAmp 9700, Roche LightCycler 96, and Thermo Scientific Arktik, vendor literature indicates maximum temperature ramp rates in the 1.5 to 5°C/sec range, sample to sample temperature uniformity of +/- 0.1 to 0.5°C, and temperature accuracy of +/- 0.25°C for sample volumes typical of standard 0.1, 0.2, and 0.5 mL centrifuge tubes and 96- or 384-well plates. Some companies have worked to improve ramp rates by optimizing thermal block design (Bio-Rad S1000: 2.5–6°C/s, 700 W), selecting high conductivity but low thermal mass block materials like silver (Eppendorf Mastercycler Pro S: 4.5–6°C/s, 950 W), scaling down system and reaction volumes (Thermo Scientific Piko: 4.5–5°C/s, 180 W [11]), reducing block size and sample count (Chai Biotechnologies OpenPCR, 1°C/s, 180 W), or reconfiguring the surface-to-volume ratio of the PCR tubes themselves (Cepheid SmartCycler: 2.5–10°C/s, 350 W [59]; Streck Philisa: 12–15°C/s [60]). Even the pioneering Idaho Technology LightCycler convective cycling design [61,62], embodied today by the Roche LightCycler 2.0 and Qiagen Rotor-GeneQ systems (15–20°C/s nominal, 1.9–3.6°C/s in-capillary, 800 W), achieves speed at the expense of power and portability. Of some 45 commercial bench-top cyclers surveyed, instrument mass ranges from 2.5 to 69.5 kg with a median of 10.5 kg. Instrument volumes range from 1.6 to 250 dm^3^ with a median of 30.4 dm^3^. Mass, volume, and power for these systems are summarized in S1 and S2 Figs.

### 1.2. Microfluidic Thermal Cycling

In contrast to commercial cyclers formatted to use disposable centrifuge tubes or sealable well plates, microfluidic thermal cycling designs are characterized by two significant differences: higher complexity (and therefore cost) of wetted components and larger surface-to-volume ratios. Cost is perhaps the most significant deciding factor when evaluating the tradeoffs between reuse and disposability, while surface-to-volume scaling determines the influence of surface chemistry artifacts on PCR performance.

#### 1.2.1. Reuse vs. Disposability

The design tension between reusability and disposability in DNA amplifying systems has long been recognized [63], and while standard laboratory practice favors rigorous PCR hygiene [51,52] and the more conservative disposable-reactor approach, many authors have reported success in cleaning and reusing PCR devices by various means. Bleach is known to be particularly effective for nucleic acid decontamination [33,64,65], but sodium hydroxide [66–69], hydrochloric acid [37,66,69], sulfuric acid [37], hydrogen peroxide [38], methanol [37,70], bromophenol blue [71], buffer [72], and even water-only flushing [73–75] have been used successfully to clean microfluidic PCR devices. Prakash and coworkers suggested stripping and re-silanizing previously treated glass PCR chips between runs to prevent carryover [68], while Obeid and team applied a more tenacious decontamination protocol, baking chips at 300 C and soaking overnight in nitric acid, to address long-term protein accumulation [73]. Designs incorporating online decontamination [33,38,72,74] are particularly suited to field-deployed or portable applications in which offline manual cleaning or automated consumable replacement would be impractical and cost-prohibitive.

#### 1.2.2. Surface Chemistry

Most microfluidic spatial thermal cyclers have the disadvantage of bringing the mobile sample into contact with significantly more surface area than stationary-sample temporal approaches. The effects of surface chemistry on PCR efficiency and inhibition in high surface-to-volume ratio systems are well known [76–84]. These effects are enhanced by the long effective transport lengths in flow-based spatial cyclers [53,74,85,86] and may be further exacerbated by the bolus mixing inherent to multiphase segmented flow in capillary and microchannel devices [87,88].

Flowing PCR mix through polymer tubing, Gonzalez and co-workers demonstrated surface-to-volume ratio-dependent but flow rate and residence time-independent adsorption of DNA and Sybr Green dye [85]. Christensen et al. showed that PCR inhibition due to Taq polymerase interactions with silicon, glass, and SU-8 can be overcome by pretreatment with bovine serum albumin (BSA) or dichlorodimethylsilane [79]. Kolari and team used real time PCR to identify PCR mix interactions with various microelectromechanical systems (MEMS) materials and further tested the effects of BSA and poly-vinyl pyrrolidone treatments [80]. Based on similar experiments, Potrich et al. confirmed that the adsorption of Taq polymerase, not DNA, was the likely source of PCR inhibition in silicon and Pyrex structures [81]. Kodzius and colleagues used a pre-amplification incubation strategy to study the inhibitory effects of a variety of materials on DNA and polymerase and also reiterated the benefits of BSA for reducing inhibition [82]. Consistent with these findings, many developers of microfluidic PCR have reported enriching the polymerase concentration of their PCR mix [77,83,84,89,90], introducing additives [77,82,91], or using silanization surface pre-treatment [68,71] to preserve PCR efficiency.

#### 1.2.3. Temporal Thermal Cycling

In the early years of MEMS and microfluidics, a number of researchers pursued rapid PCR by adapting micromachining [77,92,93] and other approaches [94] to essentially scale down conventional, often pipette-loaded, “closed batch” temporal thermal cycling methods. While the closed batch paradigm has been revisited periodically, sometimes with impressive results [95–97], it remains fundamentally limited by the difficulty of interfacing to a larger workflow without resorting to a complex and costly robotic liquid handler. In contrast, open batch or flow-through temporal cycling designs in which samples are pumped into a reactor, cycled in place, and pumped out, offer integration and automation options better suited to extra-laboratory PCR.

Flow-through temporal cyclers are most commonly heated and cooled by thermoelectric modules or a combination of resistive (Joule) heating and fan- or compressed air-cooling, but non-contact infrared (IR) and microwave heating have also been demonstrated. While microfluidic temporal cyclers typically require less power than their commercial analogs, dynamic temperature ramping generally requires more power than fixed-temperature spatial cycling (see section 1.2.4 and S2 Fig.).

**1.2.3.1. Thermoelectric Designs:** Though they tend to be power-inefficient, Peltier-based devices provide the design simplicity of both heating and cooling given a suitable heat-sink. Khandurina et al. provided an early example of thermoelectric PCR (2°C/s heating, 3–4°C/s cooling, up to 48 W) of a 3–7 μL sample in one leg of a glass capillary electrophoresis (CE) chip followed by in situ separation and fluorescence detection [98]. Another early TE-based design used two 18.1 W thermoelectric modules with a disposable polycarbonate chip to heat and cool a 40 μL sample at 7–8°C/s and 5–6°C/s, respectively [83].

In a disposable polycarbonate cassette with thermally actuated hydrogel valves, PCR, and lateral flow assay detection, Wang et al. demonstrated Peltier heating (5°C/s) with fan-assisted cooling (2.6°C/s) of an 8 μL sample [29], an approach later applied in a highly integrated, portable system for extra-laboratory applications [99]. Pursuing further integration, Jangam and team introduced a portable (20 x 28 cm footprint), cartridge-based system for detecting HIV-1 by qPCR from whole blood in low-resource settings, achieving 9.6°C/s heating and 3°C/s cooling of a 315 μL reaction [100].

In recent years, TE-based temporal cycling has been employed in a number of automated STR genotyping efforts. Hopwood and Hurth et al. introduced a cartridge-based system enabling unskilled technicians to generate sample-to-database STR genotype results in under four hours [101,102], an approach later refined and packaged into a 39.5 x 49 x 60 cm instrument [103]. Incorporating a polymer channel thermoelectric PCR module cycling 7 μL sample volumes at a rate of about 15°C/s [104], Tan and colleagues at Network Biosystems (Waltham, MA) developed a highly integrated and automated 15-locus STR genotyping system targeting “field-forward” applications [105]. The 50 kg, 67.6 x 41.9 x 58.7 cm system draws 540 W of peak power and uses a disposable 9.3 x 15.2 x 8.4 cm BioChipSet cartridge that integrates fluid reservoirs, separation gel storage, lyophilized reagents, buccal swab introduction chambers, PCR reactors, CE separation channels, and optical, pneumatic, and high voltage interfaces. With a 400 W power budget (600 W peak) consistent with conventional-scale thermoelectric cycling, the RapidHIT, a competing 81.5 kg, 73 x 71 x 48 cm instrument by IntegenX (Pleasanton, CA), automates the GlobalFiler Express STR protocol in about 90 min, though descriptions have been largely confined to trade magazines [106,107].

**1.2.3.2. Joule Heating Designs:** While resistive heating can be monolithically integrated in many lab-on-chip fabrication processes and is power-efficient compared to Peltier designs, it requires an adjunct cooling method when used for thermal cycling. The literature describes a large number of basic flow-through Joule-heated designs. Poser et al. presented an early silicon micromachined device offering nominal ramp rates as high as 80°C/s with fan cooling at 40°C/s and typical cycling power of 2.5 W per 5–10 μL reactor [108]. Using platinum heater and resistive temperature detector (RTD) elements in a silicon and glass device, Yoon et al. demonstrated 36°C/s heating and 22°C/s cooling of a 3.6 μL sample while drawing 0.6 to 1.8 W steady-state during dwells [109]. El-Ali and coworkers used an SU-8 reactor atop a glass substrate with integral platinum heaters and thermometers capable of heating at 50°C/s and cooling at 30°C/s for a 20 μL sample with dwell power under 6.4 W [84]. Liao et al. added integral poly(dimethylsiloxane) (PDMS) pumps and valves to a glass chip-based reverse transcriptase PCR (RT-PCR) system yielding 20°C/s and 10°C/s heating and cooling, respectively, of 10 μL samples with 1.8 W cycling power [110]. In a two-phase system, Wang and team demonstrated merging, mixing, and 2-step PCR with 5–250 nL droplets heated and cooled at 9°C/s and 3.5°C/s, respectively [111].

Augmenting the basic Joule-heated design, Hou et al. presented a hybrid silicon and PDMS cycler incorporating integrated valves, air cooling, and field effect sensing qPCR to achieve 2 μL qPCR ramp rates as high as 50°C/s [112]. Ferguson and coworkers presented a disposable multifunction glass and PDMS chip incorporating symmetric PCR with external heaters (ramping 2.6°C/s or less), single strand DNA generation, and electrochemical detection [31]. Bu et al. detailed a compact, integrated PCR module combining a disposable cyclic olefin copolymer chip-based reactor with optimized closed-loop forced air cooling and resistive heating to achieve ramp rates up to 5°C/s [113].

Other researchers, notably in the Mathies group at the University of California, Berkeley and at Lawrence Livermore National Lab (LLNL), made significant efforts to advance the portability and integration of Joule-heated temporal cycling devices. Woolley et al. presented one of the earliest examples of modular integration between PCR and CE, coupling a bulk micromachined, polypropylene lined, 20 μL PCR chamber with polysilicon heater (10°C/s) and fan cooling (2.5°C/s) directly to a glass CE chip [92]. With a similar lined reactor design, Northrup and team presented a battery powered, briefcase-size qPCR system using resistive heating and fan cooling to achieve 30°C/s ramping and 3.5 cycles/min with 24 W peak power [66]. Though still essentially a closed batch design, this approach was later used by Mariella in what was ostensibly the first handheld, battery-operated, multi-well PCR device [93]. With an average power of 2 W per well and yielding just under 20 s per cycle, this 1 kg design was later adapted for handheld biodetection [36].

Around the same time, Lagally et al. described a monolithically integrated, glass chip CE separation and PCR device with on-chip valving that used bond-on resistive heaters and forced air cooling (10°C/s) to achieve 20 cycle PCR in 10 min for 280 nL samples [114]. This approach was subsequently refined by including microfabricated heater and thermometer elements to achieve ramp rates as high as 20°C/s with 1 W of heater power [115], adding laser-induced fluorescence (LIF) detection [6], and adopting a radial configuration for high throughput parallel analysis of 150 nL samples [116]. Rodriguez and colleagues also demonstrated the modular integration of separately fabricated 3 μL silicon PCR reactor (1–2 W, up to 11°C/s) and glass CE chips [67].

At LLNL, Belgrader et al. presented a modular, reusable, and readily automated flow-through PCR system, fluidically similar to the RZTC, which incorporated in situ fluorescence detection, resistive heating, and forced air cooling to yield ramp rates estimated at 2°C/s (44 s/cycle) for 11 μL samples [33]. This PCR module was subsequently integrated into the deployable 170 kg, 1.44 x 0.72 x 0.88 m Autonomous Pathogen Detection System (APDS) to provide verification of immunoassay bioaerosol detection results [34].

Shortly thereafter, Liu et al. presented a further evolution of the Mathies group’s integration efforts, yielding a portable (10 kg, 30.5 x 25.4 x 10.2 cm) instrument for STR genotyping [117]. Retaining the same Joule heated (11.5°C/s) and air cooled (4.7°C/s) chip-based PCR format to amplify a 160 nL sample, the device accomplished amplification and detection in 1.5 hours while consuming 20 W. The system was subsequently tested at a realistic mock crime scene [43] and updated to include bead-based purification and clean-up for sample-in answer-out STR functionality [118,119].

**1.2.3.3. Non-Contact Temporal Cycling:** Although not as widely applied as the conductive temperature control approaches described above, non-contact heating methods offer significant benefits to speed, power consumption, and ease of integration for microfluidic cycling applications. Though configured in a closed batch format, the device presented by Pal et al. provided perhaps the earliest demonstration of microwave induction heating for fast (6.5°C/s), low-power (~1.4 W) PCR in 1 μL volumes with a free convection cooled (4.2°C/s) silicon and glass structure [120]. Later, Shaw and team presented a self-contained, modular, and readily-integrated PCR reactor using microwave heating and air impingement cooling to provide up to 65°C/s ramping of a 0.7 μL sample with 500 mW average power [121].

More widely investigated, infrared heating for PCR (10°C/s) was demonstrated by Oda et al. using 5–20 μL closed batch glass microchambers and forced air cooling (20°C/s) to yield PCR cycles as short as 17 sec or a full 30 cycle amplification in 12–14 min [122]. Terazono et al. provided another closed batch example in which a 0.9 W, 1480 nm IR fiber laser heated oil-immersed 10–30 nL droplets at up to 33.6°C/s for complete 50 cycle PCR in 3.5 min [123]. Using a similar approach, Kim and team showed selective heating of 20–100 nL droplets with a laser diode to achieve 40 cycle PCR in 6.2 min with only 30 mW power [124]. In a glass flow-through chip, Yu et al. used a 50 W tungsten lamp to cycle a 10 μL sample, realizing 40 cycles in 35 min with ~2–6°C/s heating and ~1.5–2.0°C/s cooling [69]. With a 700 mW IR laser, water-cooled fixture, and polymer flow-through chip, Saunders et al. heated (3.3°C/s) and cooled (3.9°C/s) 1 μL samples to accomplish qRT-PCR in about 1 hour [125].

One of the more extensively applied examples of non-contact cycling was provided by Easley and team, who combined interferometric thermometry with tungsten lamp heating (50 W) and forced air-cooling [126] in a design that was subsequently adapted to a glass chip-based DNA analysis system implementing extraction, amplification (550 nL reaction), separation, and detection functions in under 30 min [37]. Later IR cycling optimization yielded 25 cycles in 5 min for a 270 nL sample with heating and cooling rates of up to 23°C/s and 26°C/s, respectively [127], an approach that was later coupled with solid phase extraction in valveless chip-based systems for STR analysis [128] and RT-PCR [39] and subsequently adapted to a polymer chip format for swab-to-PCR-product STR sample preparation in under 45 min [129].

#### 1.2.4. Spatial Thermal Cycling

With a few exceptions described below, most spatial microfluidic cyclers operate in either a continuous or discontinuous (oscillating) flow mode. Continuous flow spatial cycling is characterized by a flow-path, capillary, or microchannel that repeatedly traverses a set of constant-temperature zones, producing a fixed number of thermal cycles when a sample is pumped through the device. Oscillating flow cyclers also use fixed temperature zones, but typically rely on segmented or bolus flow and dynamic, bidirectional pumping to shuttle the sample between zones. Power consumption in both spatial approaches tends to be significantly lower than similarly-scaled temporal cyclers. While continuous flow cyclers can be simpler to implement and are potentially faster than oscillating designs, they lack the flexibility to arbitrarily vary cycle count or dwell time ratios.

**1.2.4.1. Continuous Flow Designs:** Nakano et al. presented perhaps the earliest example of continuous flow PCR, routing a Teflon capillary among three constant temperature baths to provide 30 cycle amplification in as little as 18 min [70]. Another capillary-based design by Park and team, which by coincidence superficially resembles the RZTC presented here, produced cycling by flowing sample through fused silica tubing wound helically around a cylinder subdivided into discretely heated, constant temperature segments [71], an approach later adapted by others [15,130].

Kopp et al. introduced a now-canonical chip-based design in which serpentine microchannels meander over discrete heated blocks, yielding 20 cycles in as little as 90 s for a 10 μL sample [72]. In a similar design, Schneegaß and team used a silicon backplane with heaters, thermometers, and bulk etched thermal isolation voids to achieve 25 cycle PCR at 85 s/cycle for 1–10 μL oil-separated samples [78]. Using alternating wide and narrow channels, Crews et al. introduced a gradient-based “hold-less” design providing fast cooling (10°C/s) and slow heating (4°C/s) for 40 cycle PCR in under 9 min [131]. Targeting biodetection, Grover and coworkers used a clever but microfabrication-intensive multilayer 3D channel design to cycle a 20 μL reaction 30 times in 2.5–7.5 min [38].

Eschewing the more common serpentine channel and parallel heater configuration, Chen et al. presented a racetrack-shaped continuous flow design with tiled temperature zones, yielding 30 s/cycle and PCR in under 15 min [74], a design later parallelized for high throughput (15–46 s/cycle) [132] and integrated with purification, separation, and LIF detection functionalities [133]. In another highly integrated glass and PDMS design, Jha and team detailed a low power (< 3 W) continuous flow PCR device with electrochemical cell lysis, CE separation, and amperometric detection capabilities [134].

To address the problem of fixed cycle counts in continuous flow PCR, Chou et al. used an in-line peristaltic pump to recirculate a 19 μL sample through a single set of on-chip heated zones (~600 mW) yielding 1.3 min/cycle [89]. In a reusable glass chip for PCR and RT-PCR, Obeid and coworkers accomplished cycle count selection by providing outlets at intervals along the length of a serpentine reactor, yielding rates as high as 13 s/cycle [73].

**1.2.4.2. Discontinuous Flow Designs:** Oscillating flow cyclers reported to date fall into two main categories: pump-driven reciprocating linear designs and circulating designs where coordinated pumping and on-chip valves move a sample bolus around a loop through different heated zones. While positive displacement pumps are most common, sample actuation has also been accomplished by thermocapillary (Marangoni) effects in a 12 W reciprocating design [135] and by magnetically actuated ferrofluid [75] and electrokinetic pumping [136] in circulating-format devices.

Chiou and colleagues described an early reciprocating flow design in which a 1 μL capillary-bound sample was actuated between heated aluminum blocks by headspace pressure over reservoirs at the capillary ends, achieving 30 cycles in 23 min [137]. In a more readily integrated design, Cheng et al. obtained reciprocating movement and 12°C/s ramping of a 4.5 μL sample by pumping against an air bubble in a dead-end channel on a circular poly(methyl methacrylate) (PMMA) substrate with a radial temperature gradient maintained by 3.5 W of heater power [138]. Brunklaus and coworkers combined a similar dead-end channel configuration with optical droplet detection on a two-temperature polymer chip to achieve rapid cycling (10–15°C/s, 30 cycles in 6 min) and precise positioning of a 20 μL sample bolus [88]. Though not configured for sample recovery, a qPCR design presented by Frey et al. addressed bolus registration by using constricted “burst valves” in a flexible PDMS device, obtaining rapid (nominally ~0.1 s) and repeatable stepwise actuation of the 125 nL sample with an off-chip linear actuator [139].

With a design enabled by on-chip pneumatically actuated PDMS valves, Liu and team presented what is likely the first example of a circulating discontinuous flow device, demonstrating low power (455 mW) PCR with 20–30 s/cycle for a 12 nL sample [58]. A similar PDMS design by Wang et al. was integrated into a handheld unit accomplishing 30 cycle PCR in 35 min for 6 μL samples [140].

**1.2.4.3. Other Spatial Designs:** While no device presented to date substantially resembles the RZTC described here, the literature does provide examples of cycling accomplished by physically moving samples among heated zones or vice versa. Borrowing from the method originally used by Mullis et al. [1], Nagai and team manually transferred microfabricated arrays of 85 pL closed batch reactors among fixed temperature hot plates to realize 16°C/s ramping and 40 cycles in 18 min [141]. Chien and colleagues automated this approach by using a rotating arm to sweep 1 μL silicon wells among heated (9.6 W) surface zones, achieving 30 cycle PCR in 36 min [142]. Targeting RT-PCR for viral RNA amplification, Jung et al. reinvented this basic approach by rotating 1- and 3-lobed glass and PDMS chips sequentially into contact with fixed-temperature blocks, yielding nominal transitions as short as 50 ms and 34-cycle amplification in 25.5 min [143]. As part of the highly integrated and automated system described by Ritzi-Lehnert et al., this paradigm was inverted, with discs bearing fixed-temperature blocks rotated in and out of contact with stationary, multifunctional microfluidic chips to accomplish 30 cycle, 120 μL PCR in 30 min with heating and cooling rates up to 1.9°C/s and 5.4°C/s, respectively [144].

A final example of spatial cycling is provided by Advanced Liquid Logic (Morrisville, NC), which developed a compact (20.3 x 33 x 53.3 cm), cartridge-based, digital microfluidic (DMF) instrument for point of care diagnostics. Incorporating magnetic bead sample preparation, fluorescence detection, and immunoassays, the system uses electrowetting forces to actuate 300–600 nL droplets between heated zones, yielding 40-cycle qPCR in as little as 12 min with nominal ramp rates as high as 58°C/s [30,145].

While DMF approaches like these have primarily emphasized monolithic integration of different functions on the device itself [146], the design of the RZTC is driven in large part by a modular integration concept in which a central DMF platform acts as a sample distribution hub or router in a larger workflow [147], providing flexible, “impedance matched” means to interface a variety of peripheral, individually optimized sample preparation and analysis subsystems, each with their own sample format and volume requirements [148,149]. We believe that this flexible approach offers significant benefits for small scale automation, particularly in systems designed to operate outside the traditional laboratory environment.

### 1.3. The Rotary Zone Thermal Cycler Concept

**Fig 1:**
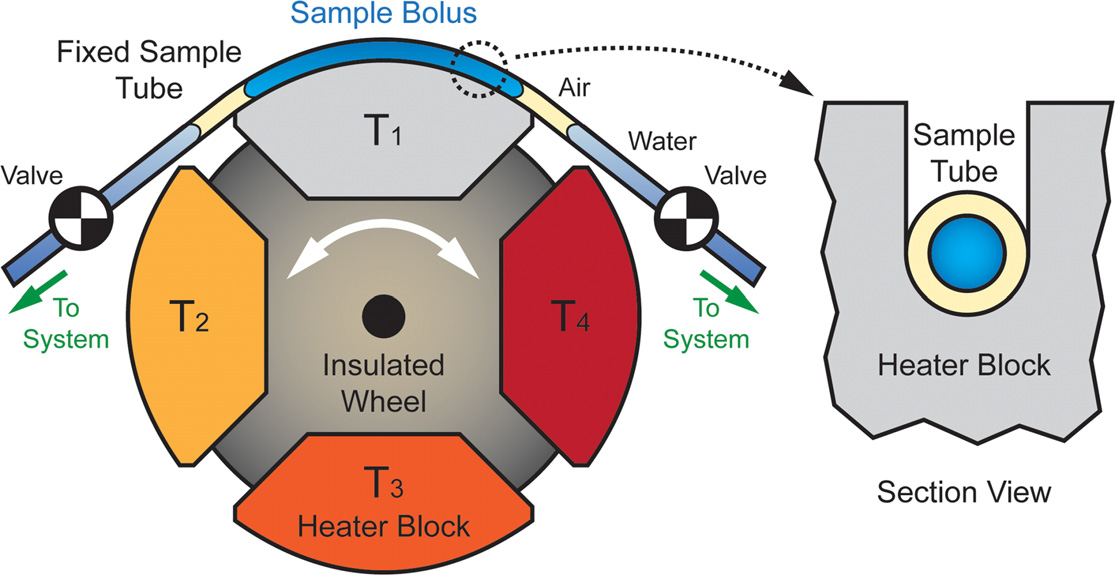
Functional schematic of the RZTC wheel. A fixed tube or capillary containing the sample to be cycled is held against temperature-controlled heater blocks, which are sequentially rotated into contact with the tube to produce the desired temperature cycling. Blocks are arranged around the wheel in order of increasing temperature. The tube rests in a groove on the outer surface of the heater block (right) and is tensioned against the block to maximize thermal coupling and sample ramp rate.

With an eye toward integrated, automated operation outside the laboratory, the RZTC capitalizes on the power efficiency of spatial cycling while maintaining the comparatively low surface-to-volume ratio of stationary-sample temporal cyclers [150]. Depicted in Fig. 1, the RZTC consists of a segmented wheel-like structure in which each segment holds a thermally conductive block maintained at a fixed temperature. A capillary or length of tubing is held in a fixed position against the wheel, contacting one heater block at a time and resting in a recessed groove that maximizes thermal contact while allowing the wheel to rotate freely as shown in the section view of Fig. 1.

In a flow-through format, the sample bolus is loaded into the reactor bracketed by air bubbles and water (as in [33]) and centered over the underlying block. Because evaporative flux and temperature transients can produce bolus movement during cycling [139], rotary shear valves are closed on the bounding water boluses to lock the sample in position. Thermal cycling is then accomplished by rotating the wheel against the fixed sample capillary to bring the preheated blocks into contact with the reactor in the desired sequence. Rapid temperature transitions are enabled by the low thermal mass of the sample and capillary and the ability to quickly and precisely actuate the wheel from one segment to the next by stepper motor. After cycling, the sample is unloaded, and the tube is cleaned in preparation for the next sample.

## Materials and Methods

### 2.1. Rotary Zone PCR Implementation

The rotary zone PCR (rzPCR) approach has two key elements: the rotary zone thermal cycler itself and the system of supporting hardware and software that enables it to function as a fully or semi-automated sample amplification module.

#### 2.1.1. Rotary Zone Thermal Cycler Design

Referencing Fig. 1, the thermal response of the RZTC sample can be understood through a simplified quasi-lumped capacitance transient analysis. The “lump” of interest is defined as the sample and capillary, convection is neglected, and the heater block is treated as a constant temperature thermal reservoir. When the wheel rotates, a block at temperature *T*_*B*_ is brought quickly into contact at time *t*_*0*_ with a sample capillary at initial temperature *T*_*SC*_, and the temperature of the capillary and sample asymptotically approach that of the arriving block according to a bounded exponential of the form:

$$T(t)=T_{B}+(T_{SC}-T_{B})e^{-(t-t_{0})/\tau }$$ 1

Where *τ* is the time constant characteristic of heat transfer between the capillary and block, a key figure of merit for RZTC design and performance. The time constant, or 70.7% rise/fall time, is conventionally interpreted in lumped capacitance analysis as the product of the thermal resistance to heat flow into or out of the lump (i.e. capillary plus sample) and the thermal capacitance of that capillary/sample combination, i.e. *τ* = *RC*.

The thermal resistance between the heater block and sample volume consists of the thermal contact resistance between the tube and block in series with the conductive thermal resistance of the capillary tube wall. While contact resistance must generally be determined empirically, it can be minimized by increasing the bearing force between the two surfaces or including conductive thermal grease and will be neglected here for simplicity. The half-cylinder radial conductive resistance [151] of the capillary wall is given by

$$R=\frac{\ln(d_{o}/d_{i})}{\pi Lk_{c}}$$ 2

Where *k*_*c*_ is the thermal conductivity of the capillary wall (W/m-K), *L* is the length of tubing held against the block, and *d*_*i*_ and *d*_*o*_ are the inner diameter (ID) and outer diameter (OD) of the tube. Thermal capacitance generally is given by:

$$C=\rho c_{P}V$$ 3

Where *ρ* is density, *V* is volume, and *c*_*P*_ is specific heat capacity at constant pressure (J/kg-K) of the thermal mass in question. Sample capillary thermal capacitance (assuming minimal internal resistance and/or long time scales) will be

$$C_{SC}=C_{s}+C_{c}=L\frac{\pi }{4}\left[\rho_{s}c_{Ps}d_{i}^{2}+\rho_{c}c_{Pc}\left(d_{o}^{2}-d_{i}^{2}\right)\right]$$ 4

Where *c* subscripts refer to the properties of the capillary tube, and *s* subscripts refer to those of the sample volume itself. Neglecting the internal thermal resistance of the sample volume, the time constant for heating or cooling the sample and capillary can be estimated as the *RC* product of equations 2 and 4, a result that is independent of length *L*.

Treating heated blocks as infinite isothermal reservoirs assumes that they are both highly conductive and thermally massive compared to the sample capillary. After an RZTC block rotates into contact with the capillary, the final equilibrium temperature (ignoring active heating) obtained by thermal circuit analysis will tend toward:

$$T_{eq}=\frac{T_{B}+T_{SC}\left(C_{SC}/C_{B}\right)}{1+\left(C_{SC}/C_{B}\right)}$$ 5

Which approaches *T*_*B*_, the initial block temperature, as the ratio of sample to block capacitance (*C*_*SC*_/*C*_*B*_) approaches zero. As *C*_*SC*_/*C*_*B*_ becomes larger, the discrepancy between *T*_*eq*_ and *T*_*B*_ increases, manifesting as an increasing temperature error (too high when ramping down or too low when ramping up) that may require additional heater power (or cooling) to return the block to its target temperature after wheel rotation.

In summary, rapid RZTC cycling is facilitated by reducing the combined sample and capillary thermal mass and by reducing block-to-sample resistance, first by tensioning the tube against the block to minimize contact resistance, then by selecting thin-wall, high conductivity tubing where possible. Temperature perturbations due to wheel rotation, sample introduction, and flushing are reduced by minimizing *C*_*SC*_/*C*_*B*_, resulting in lower steady state power consumption, but large *C*_*B*_ requires more energy to heat blocks to their set-points at startup. Because heat is lost primarily through free convection, steady state power consumption can be further reduced by shrinking wheel dimensions (i.e., surface area) or by insulating segments not engaged with the sample capillary.

#### 2.1.2. RZTC Implementation

**Fig 2:**
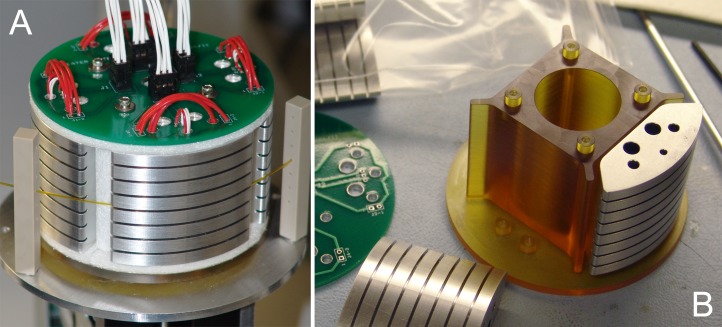
Rotary zone thermal cycler prototype. (A) Aluminum heater blocks with seven capillary grooves for parallel operation feature embedded cartridge heaters and RTDs interfaced to a printed circuit board and wire harness. Guide posts to either side of the heated zone have through-holes to maintain capillary alignment in the groove and provide options for tensioning the tube against the wheel. (B) Disassembled RZTC wheel showing insulating hub structure and heater block geometry. Axial holes in the heater block are for mounting screws (large inner pair), cartridge heaters (smaller outer pair), and RTD (smallest central hole).

Fig. 2 shows the first prototype of a four-segment RZTC. The wheel consists of a central Ultem hub (Fig. 2B), to which four aluminum heater blocks are mounted. The blocks are 3.81 cm tall (axial), 1.59 cm thick (radial), and 4.5 cm wide with a convex outer surface radius of 3.81 cm spanning 73.6 degrees of arc for a wheel diameter of 7.62 cm. The size and 55 g mass of the blocks provide thermal stability (*C*_*B*_ ~ 49.3 J/K), while the high conductivity of the aluminum enhances temperature uniformity. The curved outer surface of each block is machined with a series of 0.84 mm wide, 4 mm deep round-bottom grooves to provide good thermal contact with standard 1/32 in (0.8 mm) OD tubing and to enable parallel cycling of up to seven samples (Fig. 2A). For typical 406 μm ID, 794 μm OD fluorinated ethylene propylene (FEP) tubing, block dimensions yield a sample volume of about 4 μL.

To maximize thermal isolation, each of the four blocks is attached to a pair of small pillars on the hub (Fig. 2B) using nylon screws. As shown in Fig. 2, the gaps between the heater blocks, the hub, and the printed circuit board (PCB) are filled with 3.2 mm fiberglass felt (McMaster-Carr, Elmhurst, IL) to provide additional insulation. A computer-controlled NEMA-17 bipolar stepper motor with 26.85:1 planetary gearbox (42BYGH40M-160–4A, Phidgets, Calgary, AB), which is coupled to the RZTC hub, bi-directionally actuates the wheel from one temperature zone to the next. While not yet optimized for portability, the entire RZTC assembly including wheel, motor, and fixtures weighs 960 g.

Each RZTC block is instrumented with an embedment-type RTD (S13282PD3T36, Minco Products, Minneapolis, MN) positioned in close proximity to the radius of the capillary trenches (Fig. 2B). Axially uniform block heating is provided by a pair of symmetrically arranged, axially embedded 3.81 cm, 60W, 24V, cartridge heaters (H150–60–24–01, Sun Electric Heater Co., Salem, MA). RTDs and heaters are wired to a custom PCB as shown in Fig. 2A, which provides connections through a wire harness to a heater control box with four temperature control modules (CNi3243-C24-DC, Omega Engineering, Stamford, CT) providing independent feedback control of each heater block based on its RTD measurement. While future RZTC iterations will use an electrical slip-ring interface for heater power and temperature signals, the fixed wire harness of the current design limits unidirectional wheel rotations to 270° to avoid excessive twisting of the harness wires.

#### 2.1.3. Rotary Zone PCR System Architecture

**Fig 3:**
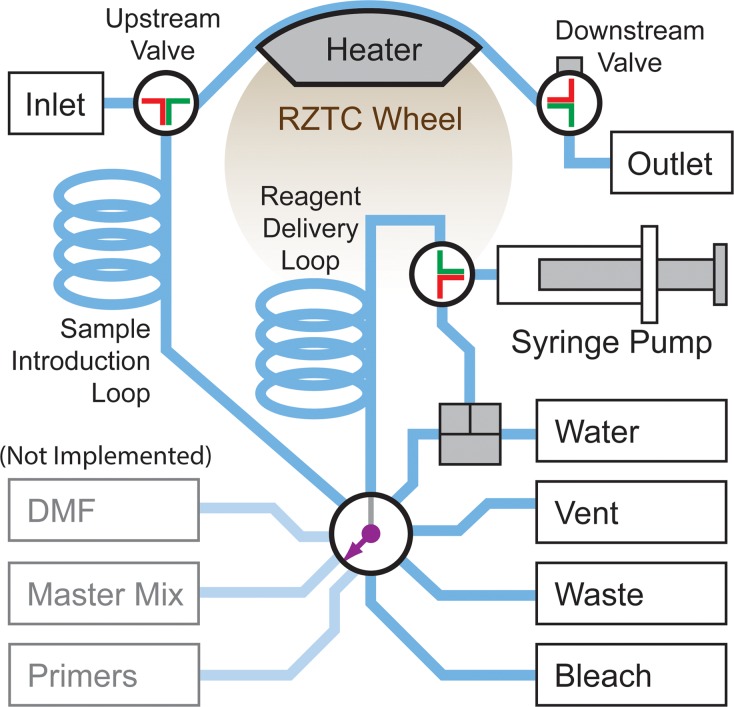
Functional fluidic schematic of the rzPCR system. A single automated syringe pump and multiport valve provide for sample loading, positioning, and unloading as well as between-run cleaning of the sample inlet and reactor tube. Upstream and downstream 3-way valves lock the sample in position over the center of the wheel during cycling.

**Fig 4:**
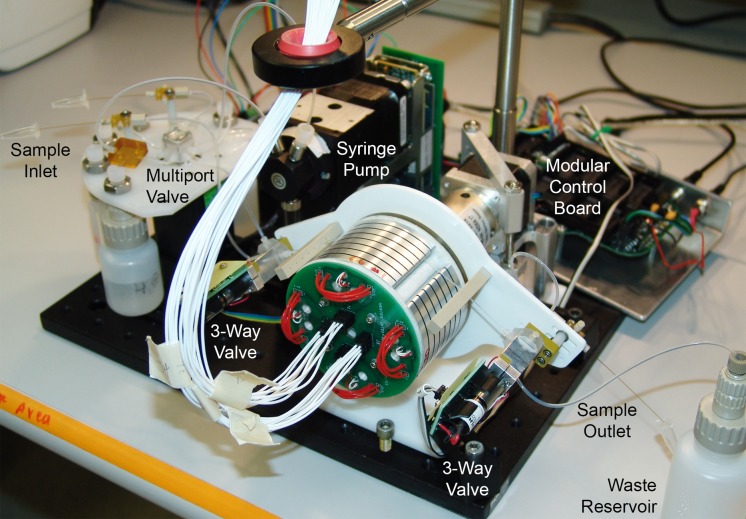
Integrated rotary zone PCR system corresponding to the schematic of Fig. 3. The wheel is oriented horizontally with the wire harness for heater power and temperature sensor measurements routed upward to the four-channel Omega controller box (Fig. 5B) on a shelf above the system. The sample capillary is tensioned against the upper part of the RZTC wheel by Sandia TubeTite connectors at the three-way valves upstream and downstream of the heated zone. Not visible in the photo are the Keyspan serial hub and RS485 converters behind the syringe pump.

**Fig 5:**
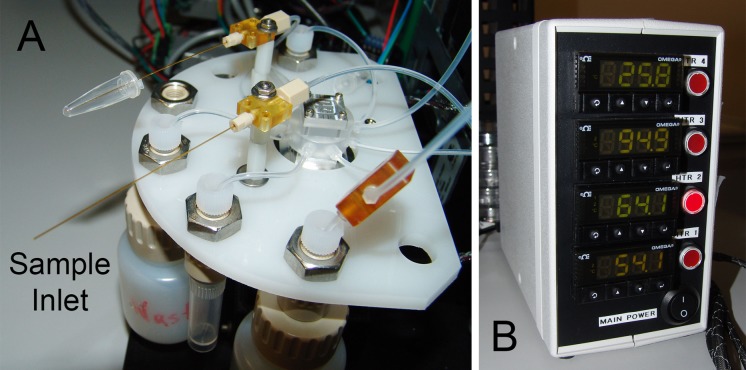
rzPCR system hardware. (A) Detail of the sample inlet and reagent distribution portion of the rzPCR system. Sample is aspirated into the capillary shown in the foreground, while the capillary in the back will deliver PCR reagents to a DMF platform in future iterations of the system. Reagent, flushing, and waste reservoirs plumbed to the central multiport valve can be readily emptied or refilled as needed. (B) Four-channel heater control box.

The fluidic architecture of the rzPCR module is shown schematically in Fig. 3, with corresponding hardware shown in Figs. 4 and 5. Sample and reagent manipulations are provided by a 500 μL syringe pump with integral 3-way valve (PSD/4, Hamilton Company, Reno, NV) and Sandia-developed low-volume multiport and 3-way rotary shear valves. To minimize opportunities for contamination and carryover, DNA-bearing sample aspirated through the inlet never moves upstream of the ~50 μL sample introduction loop, keeping the multiport valve clean. Likewise, the syringe is kept clean by preventing liquid from the multiport from moving upstream of the ~200 μL reagent delivery loop. The syringe pump is also refilled directly from the water reservoir through a bypass tee to avoid contaminants in the multiport valve. Finally, all downstream valves and tubing can be flushed with bleach and water, as discussed in section 2.2.2.1.

While not yet packaged or optimized for portability, the weight of the wheel and all major rzPCR hardware is about 1.6 kg, not counting the 1.2 kg pump and the 1 kg heater controller, both of which would be replaced in a portable version of the module. The entire module depicted in Fig. 4 (including the volume of the controller, Fig. 5B) occupies an envelope of less than 15 dm^3^.

**Fig 6:**
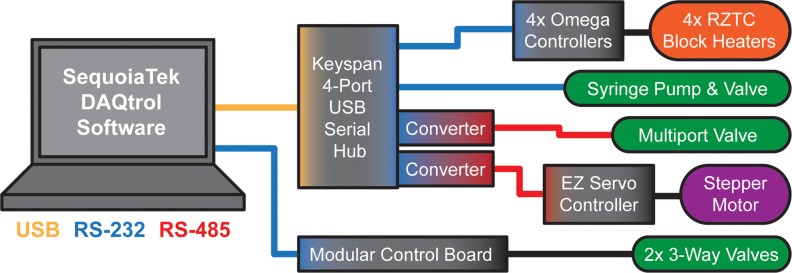
rzPCR system communication and hardware control architecture. A laptop-based DAQtrol graphical user interface (S3 Fig.) provides control and automation of the rzPCR system via pre-programmed scripts or manual control over pump, valve, heater, and data-logger operation.

As shown in Fig. 6, the rzPCR system is controlled using platform-independent SequoiaTek DAQtrol universal hardware interface and data acquisition software (www.sequoiatek.com, Logan, UT) run locally on a Windows laptop or remotely via network. The customizable DAQtrol graphical user interface (S3 Fig.) combines manual control over pump, valve, wheel, and heater operation with JavaScript-based programming that allows complex, fully automated protocols to be assembled from sequences of low level functions (e.g. refill syringe, load sample, clean sample inlet, etc.). The laptop interfaces via universal serial bus (USB) to a Sandia modular serial interface board [152] and Keyspan 4-port USB/serial interface hub (USA-49WG, Tripp Lite, Chicago, IL), which provides RS-232 serial connections to the heater control box and syringe pump as well as RS-485 serial connections (via RS485 adapters, AllMotion, Union City, CA) to the multiport valve and the Easy Servo controller (EZHR17EN, AllMotion) that drives the RZTC stepper motor.

In a typical reagent manipulation, the pump first pushes a volume of water through the reagent delivery loop and multiport to the waste reservoir, making room for reagent aspiration. The multiport valve is switched to vent, and a 3–5 μL volume of air is pulled into the reagent delivery loop to separate the pump working fluid from the reagent to be aspirated. The multiport then selects the desired reagent reservoir, and a volume is pulled into the reagent delivery loop. The multiport is switched again, upstream and downstream valves are set appropriately, and the reagent is pushed to the RZTC reactor tube and out through the module outlet. In the current implementation, the downstream valve selects between a closed position and the outlet, which is manually configured by the user for either sample or waste collection. In future iterations, the downstream valve will automatically select between sample, waste, and closed states.

As Fig. 3 indicates, automated handling of PCR reaction components (primers and master mix) has not been implemented in the current system. This functionality will ultimately be used in conjunction with a capillary-connected DMF platform [147–149] that will mix PCR reagents delivered by the rzPCR module (through the “DMF” port) with sample DNA extracted by a separate module. The PCR mix will then be aspirated into the rzPCR module from the DMF platform via the inlet. In the current scheme, PCR reactions are manually prepared at the bench and loaded through the inlet capillary shown in Fig. 5A using a semi-automated protocol.

Sample loading proceeds by first priming the reactor with water, then switching the upstream valve to the inlet position and pulling 4 μL of sample through the air-purged inlet tube (Fig. 5A). The sample, followed by 2 μL of air, is pulled just past the upstream valve, then the valve is switched and the air and sample are pushed into the reactor. The valve is switched back to the inlet and the air which preceded the sample during aspiration is pushed out until only 2 μL remain upstream of the valve. The valve is switched to the wheel and the trailing air bubble with water behind it is pushed into the reactor until the sample is centered over the heater block. Valves are then closed to isolate the reactor for cycling. After amplification, the valves are reopened, the leading water bolus is pushed out and discarded, the user is prompted to collect the sample bolus as it is dispensed, and the trailing water slug is discarded.

### 2.2. Experimental Design

Preliminary experiments to date have addressed three considerations: 1) characterizing RZTC thermal performance, 2) evaluating rzPCR operation for reproducibility and run-to-run carryover, and 3) providing initial proof of concept rzPCR amplification results for selected applications of interest.

#### 2.2.1. RZTC Characterization

To evaluate the thermal response time of the RZTC during cycling, a fine gauge K-type thermocouple (50 μm / 0.002 in dia., Omega Engineering, Stamford, CT) inserted into a length of 562 μm ID, 750 μm OD polycarbonate tubing (Paradigm Optics, Vancouver, WA) was installed and tensioned in a groove of the wheel, and the space around the thermocouple was filled with deionized water. Temperature measurements were made for the wheel transitioning with typical acceleration and velocity settings (6103.52 μsteps/s^2^, 4000 μsteps/s) between adjacent 96°C and 60°C heated blocks with ~60 s dwell times between each actuation. For comparison, equivalent measurements were performed for both a conventional bench-top cycler (Hybaid PCR Express, Thermo Scientific, Waltham, MA) with a slot cut in the housing to accommodate an identical capillary and an OpenPCR system (Chai Biotechnologies, Santa Clara, CA) with a similar groove cut in its heating block. Thermocouples were also fixed directly to the blocks of both commercial systems to capture any discrepancy between block and in-capillary measurements. Data were sampled at ~10 Hz using a custom LabView (National Instruments, Austin, TX) interface and thermocouple adapter (80TK, Fluke, Everett, WA).

During some rzPCR experiments, wheel rotation was recorded at 25 fps using a Sony DSC-F828 camera, and wheel transition times were measured by counting video frames (Premiere Elements 2.0, Adobe Systems, San Jose, CA). A digital recording power meter (Watts Up? Pro, Think Tank Energy Products, Milton, VT) was used to monitor wall power drawn by the 4-channel heater controller during initial RZTC warm-up and rzPCR amplification experiments. Also sampled at 1 Hz, RTD temperature readings output by the Omega controllers were concurrently recorded with DAQtrol.

#### 2.2.2. Rotary Zone PCR Experiments

**2.2.2.1. Between-Run Cleaning:** Because the RZTC relies on reusable rather than disposable fluidic components, decontamination between runs is a critical element of rzPCR amplification. Cleaning protocols must: 1) eliminate residual DNA from the system inlet, 2) eliminate amplified product from the reactor and downstream fluidics, and 3) eliminate cleaning residues that could inhibit subsequent PCR or degrade template DNA.

The baseline cleaning protocol (protocol A in Table 1) begins by flushing a full syringe of water through the reactor to waste. Next, air is pulled through the inlet past the upstream PCR valve to purge the sample introduction line, the valve is switched, and any aspirated liquid is pushed to waste through the wheel. Water in the syringe is flushed to the wheel to accommodate bleach that is pulled into the reagent delivery loop, pushed to the end of the inlet capillary, held for 15 seconds, withdrawn past the upstream PCR valve, and pushed onto the wheel, a process that is repeated three times. The remaining bleach and the water in the syringe are then flushed through the wheel, and an identical three-stage wash, incubate, withdraw, and discard sequence is performed with water to remove any residual bleach. Water is again pushed to the end of the sample introduction capillary, and the operator is prompted to sequentially wash the capillary by agitating its tip in vials of bleach, water, Life Technologies DNAzap (part 1), DNAzap (part 2), and water for 10–20 s each. Three 10 μL water droplets are then dispensed from the inlet to purge cleaning residues, and any remaining water is withdrawn past the valve and discarded through the wheel.

**Table 1:** Feature comparison of rzPCR cleaning protocols.

Protocol	Valve Rotation Wash	Final Inlet Flush	DedicatedWheel Wash	Inlet Wash	Bleach Use (μL)	Water Use (mL)	Time (min)
A	Yes	Yes	Full	Yes	270	4.3	23
B	No	No	Full	Yes	270	3.8	19.3
C	No	No	Shorter	Yes	270	3.8	18.4
D	No	No	No	Yes	90	2.3	14.6
E	No	No	No	No	0	1.5	6

Next, the cleaning protocol focuses on the PCR reactor itself (i.e. the “dedicated wheel wash” noted in Table 1). The wheel is actuated to position the hottest (96°C or 98°C) block against the reactor. Bleach (90 μL) is loaded into the reagent delivery loop and pushed in 30 μL increments through the heated capillary to waste at various flow rates followed by the remaining water in the syringe. A second 90 μL volume of bleach is loaded and pushed to the reactor, spanning both upstream and downstream valves, and each valve is actuated four times to assure that trace DNA in the valve interstices is neutralized. Between sets of eight valve actuations, the bleach slug is advanced 10 μL and incubated for 10 s, followed by a flush with the remaining water in the syringe. A second sequence of valve actuations interspersed with incremental bolus pushes is performed with water spanning the valves to remove residual bleach. The manual tip cleaning operation described above is repeated for the tubing at the wheel outlet, the wheel is flushed with water, and a “final inlet flush” (Table 1) is performed with 500 μL of water.

Conservative by design, protocol A provided a basis for testing progressively simpler protocols aimed at increasing sample throughput as summarized in Table 1. Protocol B resembles protocol A but eliminates the time consuming valve washing steps and final high-volume inlet flush, an operation incompatible with the eventual integration of a DMF device at the rzPCR inlet. Because the inlet cleaning operations already flush bleach and water through the reactor, protocol C abbreviates the dedicated wheel washing process with faster flow rates and shorter dwell times. Protocol D completely eliminates all dedicated wheel-washing operations and sets the RZTC to its highest temperature position at the outset of the inlet cleaning process. Lastly, protocol E eliminates the inlet cleaning operations as well, leaving a process that consists of flushing a full syringe of water through the wheel, manually cleaning the inlet capillary, flushing water again, cleaning the outlet tube tip, and flushing with water a final time. These five cleaning protocols were tested in the context of the Phusion / GAPDH amplification experiments described below.

**2.2.2.2. Rapid Single-Plex Amplification:** A protocol based on the rapid cycle-compatible Phusion high fidelity polymerase (New England Biolabs, Ipswitch, MA) was employed to evaluate the operation of the rzPCR system for single-plex PCR. A custom 2357 base pair (bp) plasmid incorporating a 362 bp human GAPDH sequence (pIDTSMAR-AMP: GAPDH, Integrated DNA Technologies, Coralville, IA) tagged with primers corresponding to the Illumina sequencing adaptors, which commonly serves as an amplification standard in our labs, was used as the template for positive control experiments. Reactions were assembled with 0.5 μL each of 10 ng/μL forward (5’-AATGATACGGCGACCACCGA-3’) and reverse (5’-CTCGTATGCCGTCTTCTGCTTG-3’) primers, 12.5 μL of 2x Phusion HF Master Mix, 11 μL of nuclease-free water and 0.5 μL of either 1.0 ng/μL GAPDH template for positive controls or nuclease-free water for no template controls (NTC) per 25 μL of reaction mix. Thermal cycling conditions corresponded to the lower end of the dwell time ranges recommended by the vendor. Following an initial 30 s denature at 98°C, 26-cycle amplifications were performed with denaturation at 98°C for 5 s, annealing at 60°C for 10 s, extension at 72°C for 15 sec, and a final elongation at 72°C for 5 min. The fourth unheated RZTC block was used for sample loading and unloading. For comparison, 26-cycle, 15 μL amplifications were also performed on the bench-top thermal cycler (Hybaid) with identical PCR mix, temperature settings, and step durations.

Amplified rzPCR samples were diluted to ~20 μL by adding 16 μL of water and separated using E-Gel EX precast 4% agarose gels with an E-Gel iBase separation system (Life Technologies, Grand Island, NY). Two to four microliter aliquots of each bench-top PCR sample and 2 μL of E-Gel 50 bp DNA ladder (P/N 10488–099V, Life Tech.) were also diluted to 20 μL for separation. Gels were imaged by an Alpha Innotech FluorChem 8900 with ethidium bromide emission filter and 302 nm excitation.

**2.2.2.3. Multiplex Short Tandem Repeat Amplification:** The PowerPlex 16 HS kit (Promega, Madison, WI) is typical of STR genotyping protocols optimized for use with conventional bench-top thermal cyclers for applications in forensic databasing and criminal casework. PowerPlex 16 HS reactions for RZTC experiments were assembled per the standard protocol, but the quantity of master mix was doubled to compensate for poor efficiency observed in preliminary experiments. Accordingly, the reaction mix consisted of 10 μL of 5X Master Mix, 2.5 μL of 10X Primer Pair Mix, and 5 μL of 0.2 ng/μL 2800M DNA template (Promega) per 25 μL reaction volume with water making up the balance. Bench-top samples (18 μL) were amplified with the Hybaid system, with minimized ramps and dwell times per the standard protocol (i.e. 120 s at 96°C hot start; 10 stage #1 cycles with denaturation at 94°C for 30 s, annealing at 60°C for 30 s, extension at 70°C for 45 s; 22 otherwise identical stage #2 cycles with denaturation at 90°C; and final elongation at 60°C for 30 min). rzPCR cycle times and temperatures were identical, but because the protocol requires five temperatures, block 3 of the RZTC was ramped in real time from 96°C to 90°C after the initial hot start incubation. Each sample run was preceded by cleaning protocol B as described above, and samples were processed in order: NTC, 2800M positive control, NTC. Amplified rzPCR samples were diluted to ~20 μL with 16 μL of water, and 4 μL aliquots of bench-top samples and 50 bp ladder were likewise diluted to 20 μL. E-Gel separations were performed as described above.

**2.2.2.4. Second Strand cDNA Synthesis:** Motivated by a biosurveillance application in which the genetic content of pathogen-infected blood specimens collected in the field is stabilized for transport to a central lab through conversion of RNA to cDNA, a third set of experiments was undertaken to demonstrate the utility of the rzPCR system for second strand cDNA synthesis. Human whole blood samples were purchased from ProMedDx (Norton, MA; http://www.promeddx.com). These samples were collected by independent investigator sites, from subjects who provided written informed consent. The investigator site assigned each sample an ID number for de-identification purposes, and then ProMedDx re-assigned each sample a unique lot number in a manner that prevents identification of the investigator site by those outside of ProMedDx. These pre-existing, de-identified samples were considered exempt from the U.S. Federal Policy for the Protection of Human Subjects, or Common Rule (U.S. Code of Federal Regulations reference for the Department of Energy is 10 CFR 745), such that our use of them required only notification of Sandia's Institutional Review Board (IRB00001150).

Total RNA extracts from human whole blood obtained by standard bench-top methods were fragmented using the NEBNext kit (New England Biolabs, Ipswitch, MA), following the manufacturer's instructions. First strand cDNA was synthesized from the fragmented RNA using the Peregrine method [153]. Benchtop and rzPCR second-strand cDNA synthesis reactions were assembled using 25 μL FailSafe PreMix E PCR Buffer, 1 μL adapter / primer 1, 1 μL barcode / index primer 2, 1 μL FailSafe PCR enzyme (2x the standard concentration), 12 μL water, and 10 μL first strand cDNA products per 50 μL reaction volume (all reagents from Epicentre, Madison, WI). In both the bench-top and rzPCR systems, the reactions (18 μL and 4 μL, respectively) were initially denatured at 95°C for 60 s, followed by 10–18 cycles of denaturing at 95°C for 30 s, annealing at 55°C for 30 s, and extending at 68°C for 3 min, and a final extension cycle at 68°C for 7 min. Second-strand cDNA products were assessed with respect to size and yield using a BioAnalyzer 2100 (Agilent Technologies, Santa Clara, CA) and 2100 Expert software in High Sensitivity DNA Assay mode.

## Results and Discussion

### 3.1. Thermal Performance & Characterization

#### 3.1.1. Wheel Rotation and Sample Ramp Rates

The temperature ramping performance of the RZTC comprises the nominal ramp time associated with wheel rotation plus the sample response time discussed in section 2.1.1. Given the asymptotic nature of the sample response (Equation 1), results below are couched in terms of the time required to approach within 1°C and 0.1°C of the final temperature. A less intuitive but less arbitrary comparison is afforded by the time constant, *τ*.

Wheel rotation rates determined by video frame counting were found to be consistent with those calculated based on the Easy Servo acceleration and velocity settings, yielding one-, two-, and three-block (nominal) transition times of 1.32, 1.99, and 2.66 s, respectively. S1 Section and associated S4 Fig. provide a more detailed discussion of the effect of wheel rotation and sample geometry on temperature history, while S1 Video shows video of the RZTC in operation.

**Fig 7:**
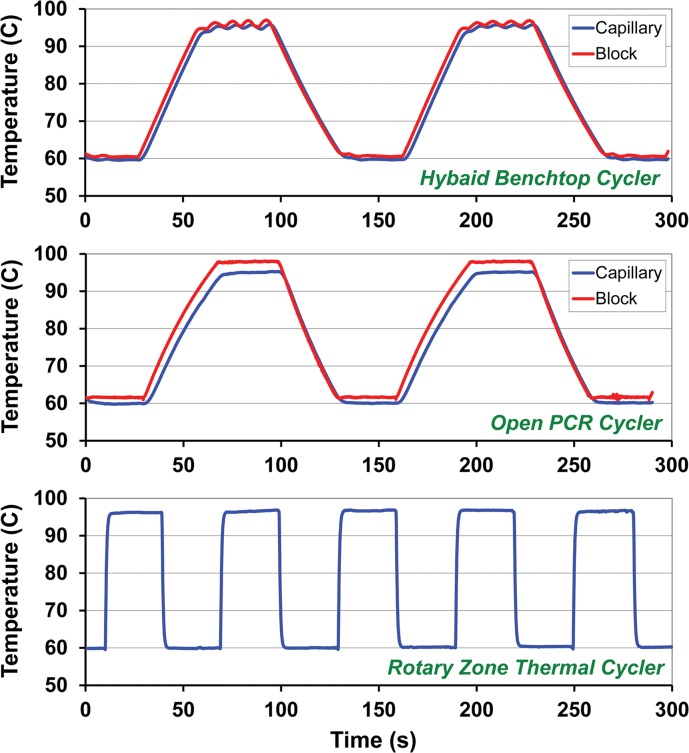
Thermal cycling performance comparison for three thermal cycler designs between 60°C and 96°C. Conventional Peltier based bench-top and hybrid Peltier/forced air OpenPCR thermal cyclers display significantly slower temperature ramp rates than those obtained by the stepper motor actuation of the RZTC.

**Fig 8:**
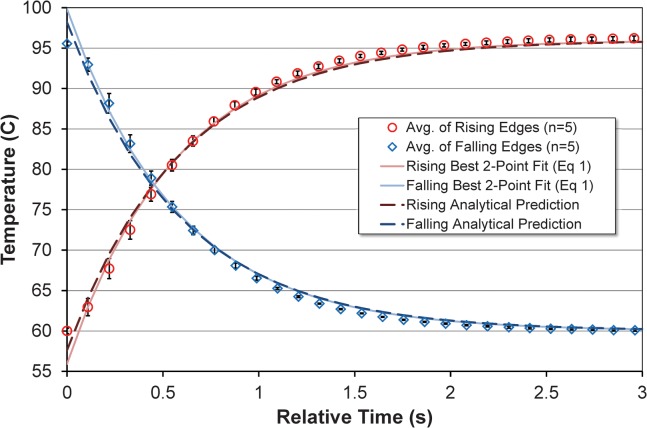
Comparison of average thermocouple-measured rise and fall times derived from the five cycles depicted in Fig. 7 with a parametrically fitted bounded exponential curve of the form of Equation 1, and response curves predicted analytically a priori from Equations 1, 2, and 4.

Fig. 7 shows direct in-capillary thermocouple measurements for bench-top, OpenPCR, and RZTC operating between 60 and 96°C, with direct block measurements included for the commercial systems. For the five RZTC cycles shown, rising and falling data were baseline-corrected, time-shift aligned, and averaged as shown in Fig. 8. For both average curves, Equation 1 was solved at two points to yield a two-parameter (*τ* and *t*_*0*_) fit iteratively optimized to maximize R^2^ over the range shown. Rising and falling time constants are 0.564 s and 0.575 s, respectively. Thermocouple-derived average rise and fall times and time constants are summarized in Table 2.

**Table 2:** Measured and analytically predicted transitions between 60°C and 96°C for samples in polycarbonate (PC) and FEP tubing with and without a thermocouple (TC) present.

Case	Sense	End T(°C)	Time (s)	Sample Ramp Rate (°C/s)	*τ* (s) [R^2^ value]
Measured PC with TC(n = 5)	Heat	95	1.78 ± 0.09	+19.7	0.564 [0.99188]
		95.9	2.49 ± 0.42	+14.4	
	Cool	61	1.87 ± 0.01	-18.7	0.575 [0.99218]
		60.1	2.92 ± 0.43	-12.3	
Analytical PC with TC	Heat	95	2.11	+16.6	0.590 [0.99257]
		95.9	3.47	+10.4	
	Cool	61	2.11	-16.6	0.590 [0.99383]
		60.1	3.47	-10.4	
Analytical FEP no TC	Heat	95	5.73	+6.1	1.598 [N/A]
		95.9	9.41	+3.8	
	Cool	61	5.73	-6.1	
		60.1	9.41	-3.8	

Time constants for measured data are derived based on 2-parameter fit of Equation 1, while predicted time constants are derived a priori from Equation 2 and 4.

While the fitted function in Fig. 8 shows fair correspondence across the range indicated, deviation of the data from the ideal bounded exponential, particularly early in the transition, likely reflects the fact that block-to-block transitions are not ideal step functions. As the data suggest, heating appears to occur slightly faster than cooling, perhaps due to heat produced by the friction of the wheel against the sample capillary during rotation. Including the typical 1.32 s single-block wheel rotation time, the total time required for a 35 degree C temperature transition (e.g., 60 to 95°C or 96 to 61°C) is about 3.1 s or 11.3°C/s. For comparison, the OpenPCR and bench-top measurements in Fig. 7 yield transition times of about 30 s, or about 1.2°C/s.

Using the analytical methodology of section 2.1.1 (Equations 1, 2, and 4), thermal RC time constants were also calculated a priori based on geometry and material properties for both the polycarbonate tubing of the thermocouple measurements and the FEP tubing used during rzPCR experiments, with results shown in Table 2. Fig. 8 shows the rising and falling curves predicted by the calculated time constant for the polycarbonate case, time shifted (*t*_*0*_ = 0.038 s rising, *t*_*0*_ = 0.035 s falling) to maximize R^2^. Since the time constant predicted a priori for the polycarbonate case is less than 5% larger than the measured value, the time constant predicted for the FEP tubing case (1.6 s) is also likely to be both reasonably accurate and slightly larger than the actual value. One potentially significant difference between the two cases is that the larger OD of the FEP tube may yield better thermal contact to the walls of the heater block groove than the smaller polycarbonate tube, further supporting the idea that the predicted FEP response may be somewhat overestimated.

#### 3.1.2. Power Consumption & Temperature Stability

**Fig 9:**
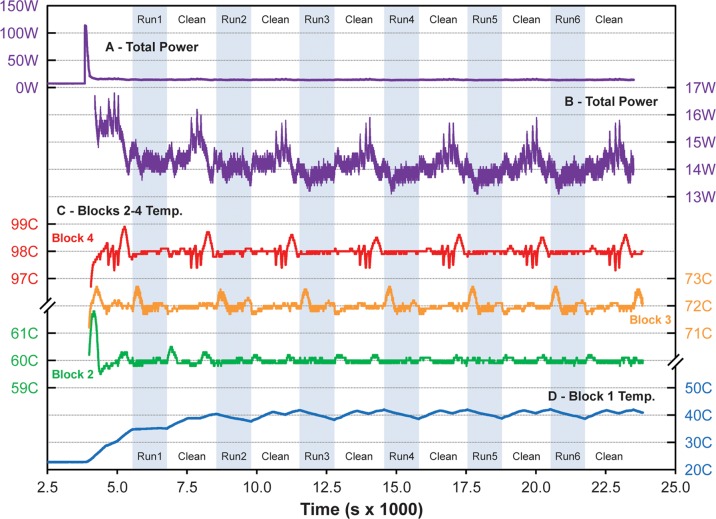
RZTC power and temperature history from startup for six Phusion rzPCR experiments with thermal cycling intervals indicated by the shaded bands. (A) Total (wall) power drawn by the heater control box during initial idle, warm-up, and cycling. (B) Detail of power fluctuations during cleaning, sample load, amplification, and sample unload. (C) Temperature fluctuations for heater blocks 2–4 about their set-points at 60°C, 72°C, and 98°C. (D) Temperature evolution of unheated block 1 over time.

Fig. 9 shows the temperature and power history from initial wheel startup through seven identical cleans and six identical GAPDH amplification experiments as discussed in sections 2.2.2.2 and 3.2.1. Room temperature was 22.6 ± 0.12°C based on average initial block RTD measurements. The initial power baseline of Fig. 9A shows that the four-channel heater control box, which is not optimized for low power operation, draws 7.29 ± 0.04 W with the heaters off. At startup, simultaneous activation of all three heaters produces an initial spike with peak power of 113.9 W and integrated energy of about 4.2 W-hr. The 60, 72, and 98°C blocks stabilize at their set-points after about 15 min, while unheated block 1 gradually drifts to around 40°C after about 120 min, then deviates systematically ± 2°C about this temperature due to positional changes in the rate of free convection as the wheel rotates. For the current design, this ~60°C temperature differential likely represents the maximum that can be sustained between adjacent wheel segments in still air. Based on the rotation times above, this implies a maximum nominal RZTC ramp rate of about ± 44°C/s as presently configured.

After the initial startup transient, overall power consumption during cycling and cleaning operations averages 14.2 ± 0.42 W, with about 7 W of that total corresponding to the heater power required to maintain the temperature of the three heated blocks. Across the six amplification experiments shown, the average deviations and maximum temperature excursions from the set-points of the three heated blocks are-0.03 ± 0.05°C with maximum deviations of +0.1 and-0.2°C for block 2 (60°C, +0.08 ± 0.22°C between +0.7 and-0.3°C for block 3 (72°C), and-0.03 ± 0.06°C between +0.1 and-0.2°C for block 4 (98°C). Larger temperature fluctuations and corresponding power spikes are associated with either water flushing during cleaning or convective heat transfer changes due to wheel position. The average time required for 26-cycle amplification was 21 minutes, while between-run unloading, cleaning (protocol A), and reloading averaged 25 ± 10 min, with variability due to the manual portions of these operations.

During thermal cycling, slight (1–2 mm) bidirectional displacements of the sample bolus are observed in response to wheel actuation, likely due to the asymmetric temperature transients imposed on the adjacent air bubble separators. These displacements average out over each full thermal cycle, so the bolus remains centered. Stable bubble formation, like that noted by Wang [29], is also observed within the sample bolus after exposure to high-temperature steps, likely due to outgassing from the reaction mix. While these bubbles do not significantly displace the sample bolus, bubbles that span the capillary diameter could limit transport of PCR components within the bolus and yield inhomogeneous, inefficient, or biased amplification. Bubble formation can likely be minimized by either degassing reagents or pressurizing the reaction volume prior to cycling.

**Fig 10:**
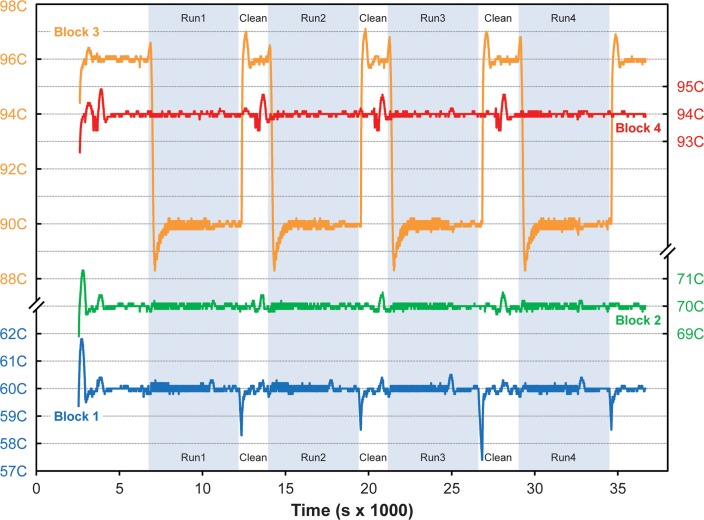
4-block RZTC temperature history from startup through a series of four PowerPlex 16 HS runs (shaded) with unloading, cleaning, and loading operations between them. Block 3 is switched in real time between 96°C hot start and 90°C cycling set-points.

Fig. 10 shows RZTC block temperatures recorded during a series of the PowerPlex 16 HS STR amplification experiments described in sections 2.2.2.3 and 3.2.2. Here, all four blocks are temperature controlled, and block 3 is switched from its initial 96°C “hot start” setting to 90°C in real time during the rzPCR protocol. As the figure shows, this transition results in a substantial undershoot of the target temperature, but block 3 stabilizes at 90°C after about 18 min (subject to future control optimization), in time for the start of stage #2 cycling (90°C, 60°C, 70°C). As implemented with the rzPCR system, 32 cycles required 91 min on average, while between-run unload, clean (protocol B), and loading operations required about 33 ± 6 min total.

Due to the length of the STR runs, complete power measurements (not shown) were only recorded for the first two experiments. Baseline power of the four channel heater control box was 7.22 ± 0.08 W, and activation of the four heaters produced an initial spike like that shown in Fig. 9A with a peak of 148.8 W and total integrated startup energy around 4.9 W-hr. Average operating power over the span of 5000 to 25000 sec indicated in Fig. 10 was 16.12 ± 1.03 W, with localized 30 W peaks associated with reheating block 3 from 90 to 96°C and fluctuations during cleaning representing the main deviations from the 16 W baseline.

While overall temperature stability during STR cycling appears to be good, a larger temperature rise of 0.5–0.8°C is observed in the block 3 temperature trace shortly after the start of cycling and just prior to the change of set-point from 96°C to 90°C. The rise appears to be an overshoot due to increased heater power (not shown) applied in response to the rotation of the 96°C block against the 60°C capillary at the start of the run. The result is a block 3 temperature error during the initial 120 s hot start phase averaging +0.42 ± 0.19°C with maximum deviations of +0.8°C and -0.0°C relative to the set-point. Following the startup transient, the average temperature error on block 3 about 90°C is-0.02± 0.10°C with maximum deviations of +0.2°C and -0.4°C. Average errors for blocks 1, 2, and 4 were 0.00 ± 0.10°C, 0.00 ± 0.06°C, and 0.01 ± 0.06°C, respectively, with maximum excursions of +0.5°C to -0.3°C, ±0.2°C, and +0.2°C to -0.3°C, respectively. Again, larger variations during the cleaning and loading phases correspond either to the reheating of block 3 or to thermal perturbation due to water flushing, mainly affecting blocks 1 and 4.

### 3.2. Rotary Zone PCR Results

#### 3.2.1. GAPDH Single-Plex and Cleaning Experiments

**Fig 11:**
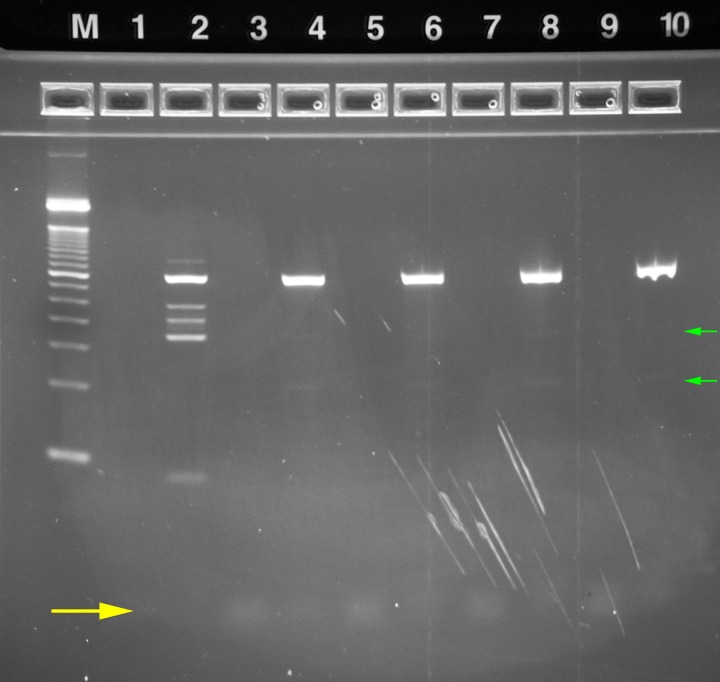
E-Gel image showing alternating positive control and no template control 26-cycle GAPDH / Phusion experiments. Lanes (M) 50 bp ladder, (1) Bench-top NTC, (2) Bench-top positive control, (3, 5, 7, 9) rzPCR NTC, (4, 6, 8, 10) rzPCR positive control. The yellow arrow indicates the position of primer bands, while green arrows indicate faint nonspecific bands in the rzPCR positive control lanes. Streaks and smudging are post-separation handling artifacts.

Fig. 11 shows the result of sequential positive and negative (NTC) 26-cycle rotary zone PCR amplifications performed with cleaning protocol A executed between each run. The first two amplified sample lanes show bench-top negative and positive results for comparison. rzPCR-amplified bands in lanes 3–10 show consistent amplification of the GAPDH target at 362 bp and consistently clean negatives with no evidence DNA carryover from the preceding positive runs. While highlighting rzPCR reproducibility, this result suggests that cleaning protocol A is effective in both preventing run-to-run contamination and eliminating residual bleach carryover, which could inhibit amplification in subsequent runs. Prominent nonspecific amplification bands in the bench-top positive lane and very weak nonspecific bands in the rzPCR positive lanes suggest that further optimization of amplification conditions in each format would be beneficial.

**Fig 12:**
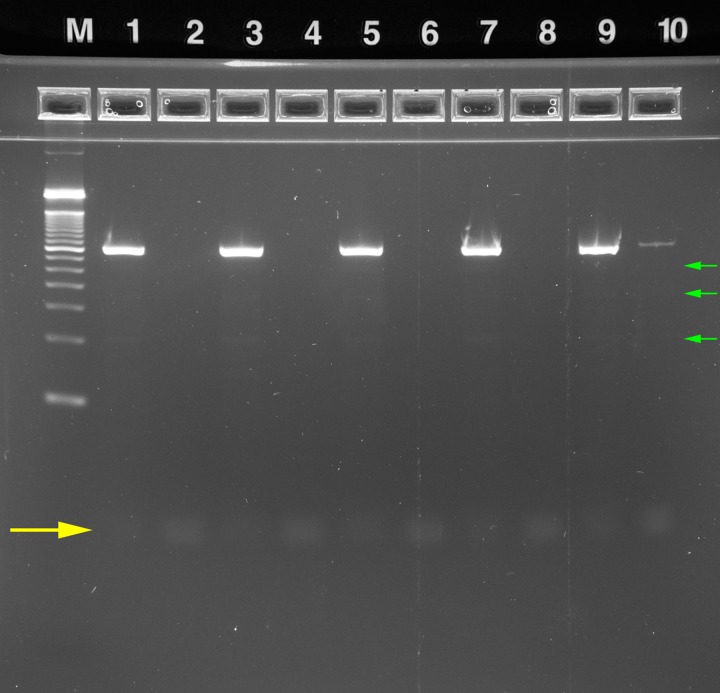
E-Gel image showing sequential 26-cycle Phusion / GAPDH cleaning and decontamination experiments. Odd numbered lanes are identical positive controls preceded by cleaning protocol A. Lanes (M) 50 bp ladder, (2) NTC preceded by cleaning protocol A, (4) NTC preceded by cleaning protocol B, (6) NTC preceded by cleaning protocol C, (8) NTC preceded by cleaning protocol D, (10) NTC preceded by cleaning protocol E. The yellow arrow indicates the position of primer bands, while green arrows indicate faint nonspecific bands in the rzPCR positive control lanes.

While cleaning protocol A provided an effective baseline, its length and excessive consumption of water make it less than ideal for portable or extra-laboratory PCR. Fig. 12 shows the results of a series of 26-cycle Phusion / GAPDH experiments evaluating the efficacy of the progressively simpler cleaning protocols summarized in Table 1. Here, all positive control experiments were preceded by cleaning protocol A, but negative experiments received different pre-run cleaning treatments. Lane 4 shows an NTC in which valve actuations and the final large volume (DMF-incompatible) inlet flush were eliminated (protocol B). Lane 6 shows the result of compressing downstream wash times (protocol C). Lane 8 shows the result of a pre-NTC clean in which dedicated downstream wash steps were eliminated altogether, with flushing provided solely by the bleach and water used to clean the sample inlet and tubing upstream of the reactor (protocol D). Protocol D also immediately set the wheel to its 98°C position so all effluent from the inlet cleaning process passed through the preheated reactor. Lastly, lane 10 shows the result of performing only manual inlet capillary tip cleaning operations and flushing the reactor with three full syringe volumes of water (protocol E). Of all the variations tested, only protocol E yielded any evidence of DNA carryover between sequential positive and negative runs as shown by the faint GAPDH band visible in lane 10. While additional repetitions and more sensitive detection (e.g. qPCR) will be needed to rigorously validate the effectiveness of the rzPCR cleaning and reuse paradigm, these encouraging initial results suggest that further simplification, optimization, and automation could reduce cleaning times to 5–10 min or less.

#### 3.2.2. STR Multiplex Experiments

**Fig 13:**
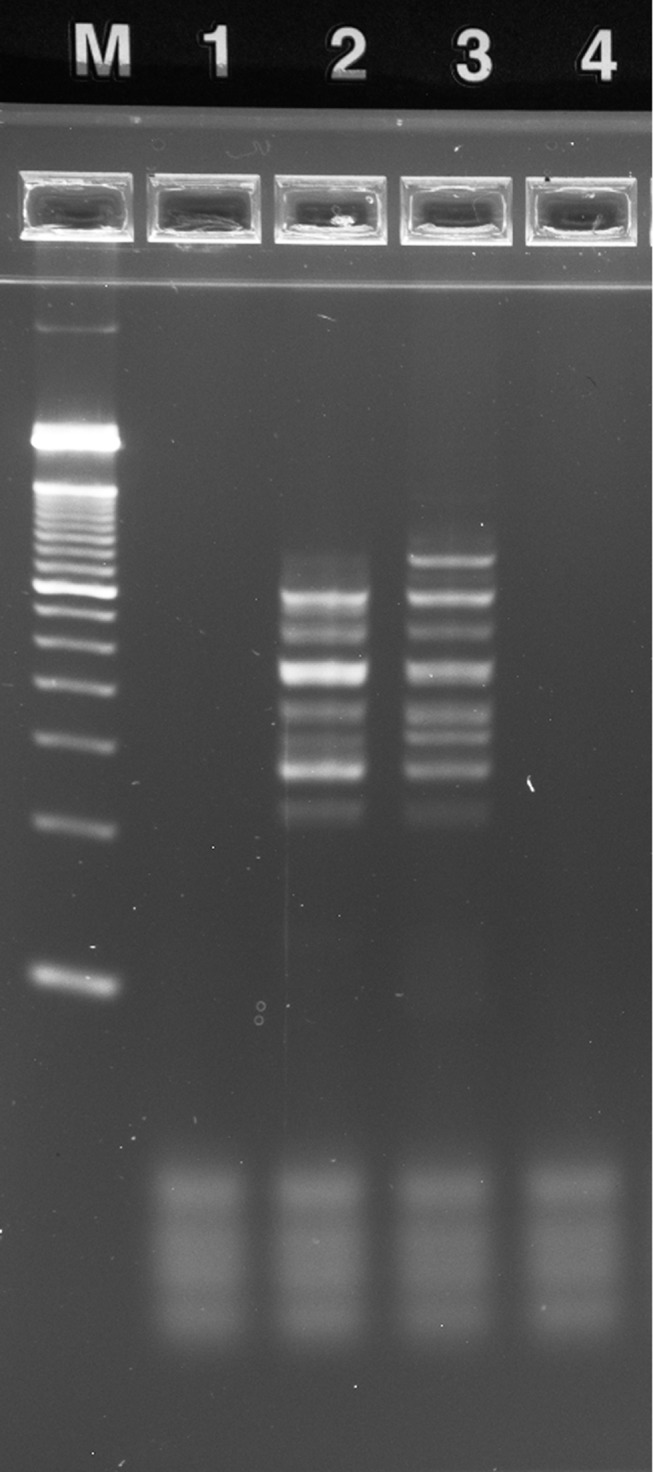
E-Gel image showing PowerPlex 16 HS amplification results. Lanes (M) 50 bp ladder, (1) Bench-top NTC, (2) Bench-top positive control 2800M template, (3) rzPCR positive control 2800M template, (4) rzPCR NTC.

Fig. 13 shows amplification results for a set of 32-cycle PowerPlex 16 HS experiments in which cleaning protocol B was run between each rzPCR sample. Here, lane 1 is a bench-top NTC, lane 2 is a bench-top positive control with 40 pg/μL of 2800M template, lane 3 is the corresponding rzPCR positive, and lane 4 shows the subsequent rzPCR no template control. While band intensities suggest that the rzPCR amplification is somewhat less efficient than the bench-top approach, peak biasing across the multiplex appears to be more pronounced in the bench-top sample. These differences could be a function of the disparate surface-to-volume ratios between the two systems, or they may indicate that the Master Mix (polymerase) enrichment that benefits the microfluidic reactor adversely affects performance in the bench-top format. Also, because the PowerPlex 16 HS system is not optimized for rapid cycling per se, ramp rate differences between the two systems may be a factor. Despite the inconsistencies, however, band intensity and definition in the rzPCR samples compare favorably to those of the bench-top result, with no indication of pronounced band broadening, smearing, or nonspecific amplification.

#### 3.2.3. Second Strand Synthesis Experiments

**Fig 14:**
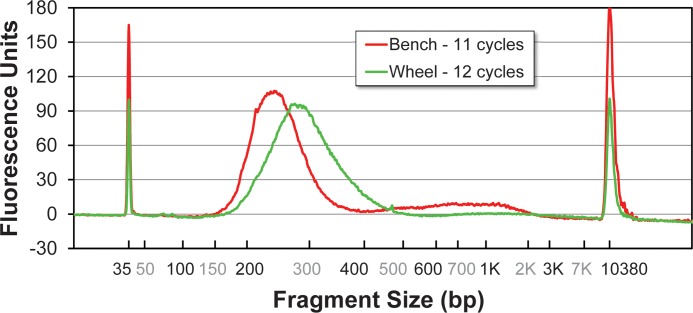
BioAnalyzer data from analysis of second-strand cDNA products generated by the bench-top and rzPCR systems. Note that rzPCR reactions were run for an additional cycle (12 cycles total, as compared to 11 cycles for the bench-top system).

Fig. 14 presents BioAnalyzer data from second-strand cDNA synthesis products generated by the bench-top and rzPCR systems. The rzPCR method generated ~2-fold lower yields, such that comparable fluorescence-intensity peak heights were achieved only after an additional reaction cycle (12 cycles total, as compared to 11 cycles for the bench-top system). However, rzPCR generated slightly larger cDNA products, averaging ~280 bp (as compared to ~240 bp for bench-top products). Taken together, these results suggest that the rzPCR system favored enzyme processivity at the expense of yield, perhaps as a result of the differing surface-to-volume ratios and/or ramp rates relative to the bench-top system. We also found that the rzPCR system generated fewer inappropriately large cDNA products (≥500 bp) resulting from a primer concatenation runaway effect [153]. This trend was observed when comparing reactions of identical cycle numbers (e.g., 12 each for bench-top and rzPCR), and even when the rzPCR reactions were subjected to an additional cycle (e.g., 11 cycles for bench-top, 12 cycles for rzPCR, data not shown). These results, too, are consistent with the idea that the rzPCR system favors enzyme processivity, generating cDNA products of higher quality (more full-length products and few primer-concatenation products) though at slightly (~2-fold) lower yield.

## Conclusion and Future Work

In this article, we have introduced the rotary zone thermal cycler, a novel wheel-based system providing rapid cycling of microliter-scale samples in a power-efficient and readily automated capillary microfluidic format. In its current implementation, the RZTC has enabled cycling among up to four temperature zones with nominal ramp rates as high as 44°C/s and maximum temperature differentials between adjacent zones of about 60°C. After an initial 3–5 min warm-up period, which consumes less than 5 W-hr, the RZTC requires less than 10 W of continuous heater power to maintain block temperatures in still room temperature air.

Beyond evaluating the thermomechanical performance of the RZTC, we have demonstrated its operation in the context of a highly integrated rotary zone PCR system. Fully automating most sample manipulation, cycling, and decontamination functions, the rzPCR module demonstrates the potential benefits of the RZTC design for molecular biology automation and sample preparation workflows in or outside the laboratory. Preliminary experiments have shown the utility of the rzPCR system for rapid single-plex DNA amplification, multiplex short tandem repeat amplification, and cDNA synthesis. In pursuit of robust, reusable operation, we have also demonstrated the effectiveness of automated bleach-based between-run cleaning protocols requiring as little as 15 minutes, a number that can likely be reduced through future optimization. In combination with its high level of automation and low power requirements, the minimal logistics burden of the rzPCR suggests that future RZTC-based systems could effectively address deployable, portable, or low-resource applications where regular servicing and resupply of specialized consumables are impractical.

In the near term, future work will focus on fully integrating the prototype rzPCR module with a digital microfluidic platform to enable fully automated end-to-end sample preparation, thermal cycling, and between-run cleaning. Additional experiments will refine, optimize, and rigorously validate decontamination and cleaning protocols while reducing cleaning times to increase sample throughput. The use of the rzPCR syringe pump to pressurize the reaction tube and mitigate sample outgassing will also be explored. Lastly, we will seek to take advantage of the multi-lane design of the RZTC for simultaneous, parallel sample processing.

In future RZTC design iterations, optimization of the actuation motor, heater block geometry, and wheel dimensions combined with the use of a free-rotating electrical slip ring in place of the current wire harness would allow for faster temperature transitions, reduced size, and increased power efficiency. Advanced development efforts will also address long-term reliability, particularly with respect to friction wear due to the motion of the wheel against the reactor tube. While thousands of leak-free wheel rotations have been logged to date using the same FEP capillary, high-cycle testing will be necessary to identify a capillary replacement interval and explore options to minimize wear. The relatively slow asymptotic in-capillary temperature response of the current RZTC design could also be improved by incorporating higher temperature preheating and lower temperature precooling blocks at opposite sides of each larger RZTC dwell block to accelerate ramping during wheel rotation, a spatial analog of the temporal overshoot/undershoot method used by Bu [113]. Additionally, the integration of in situ fluorescence excitation and detection could enable the rzPCR system to provide qPCR functionality, yielding a flexible tool for quantitation and detection as well as amplification. Finally, the suitability of rzPCR-type systems for extra-laboratory operation will ultimately need to be demonstrated by testing in realistic operating conditions with reagents that are lyophilized or otherwise environmentally stabilized.

## Supporting Information

S1 Fig Size and weight comparison of commercial thermal cycling instruments, the rotary zone PCR system, and select PCR-enabled systems with published mass and volume specifications. (TIF)

S2 Fig Comparison of ramp rate and power consumption for commercial and microfluidic thermal cycling systems. (TIF)

S3 Fig DAQtrol graphical user interface (GUI) for operating the rzPCR system. Automated, script-based commands are selected and executed in the window at left, while manual control over system components and temperature data acquisition are provided by the windows at right.
(TIF)

S4 Fig Plot of generalized wheel position and corresponding temperature history experienced by the sample during wheel rotation for the stepper motor control parameters used in this work, where t = 0 is the start of rotation. Solid lines show the progression of the underlying heater blocks relative to a point at the center of the fixed sample bolus as a function of time for one-, two-, and three-block transitions. Dashed lines show the temperature history experienced concurrently by points at the leading and trailing end of the same sample bolus.
(TIF)

S1 Section Wheel Rotation and Sample Temperature History. (PDF)

S1 Video Video sequence showing the operation and real-time (1x speed) rotation of the RZTC during a complete PowerPlex 16 HS amplification cycle. Dwell times between rotations are shown at 5x speed.
(MPG)
