# Supplementary figures and images for: The Rotary Zone Thermal Cycler: A Low-Power System Enabling Automated Rapid PCR
Source: PLoS One. 2015 Mar 31;10(3):e0118182. doi: 10.1371/journal.pone.0118182 (PMC4380418; doi:10.1371/journal.pone.0118182)

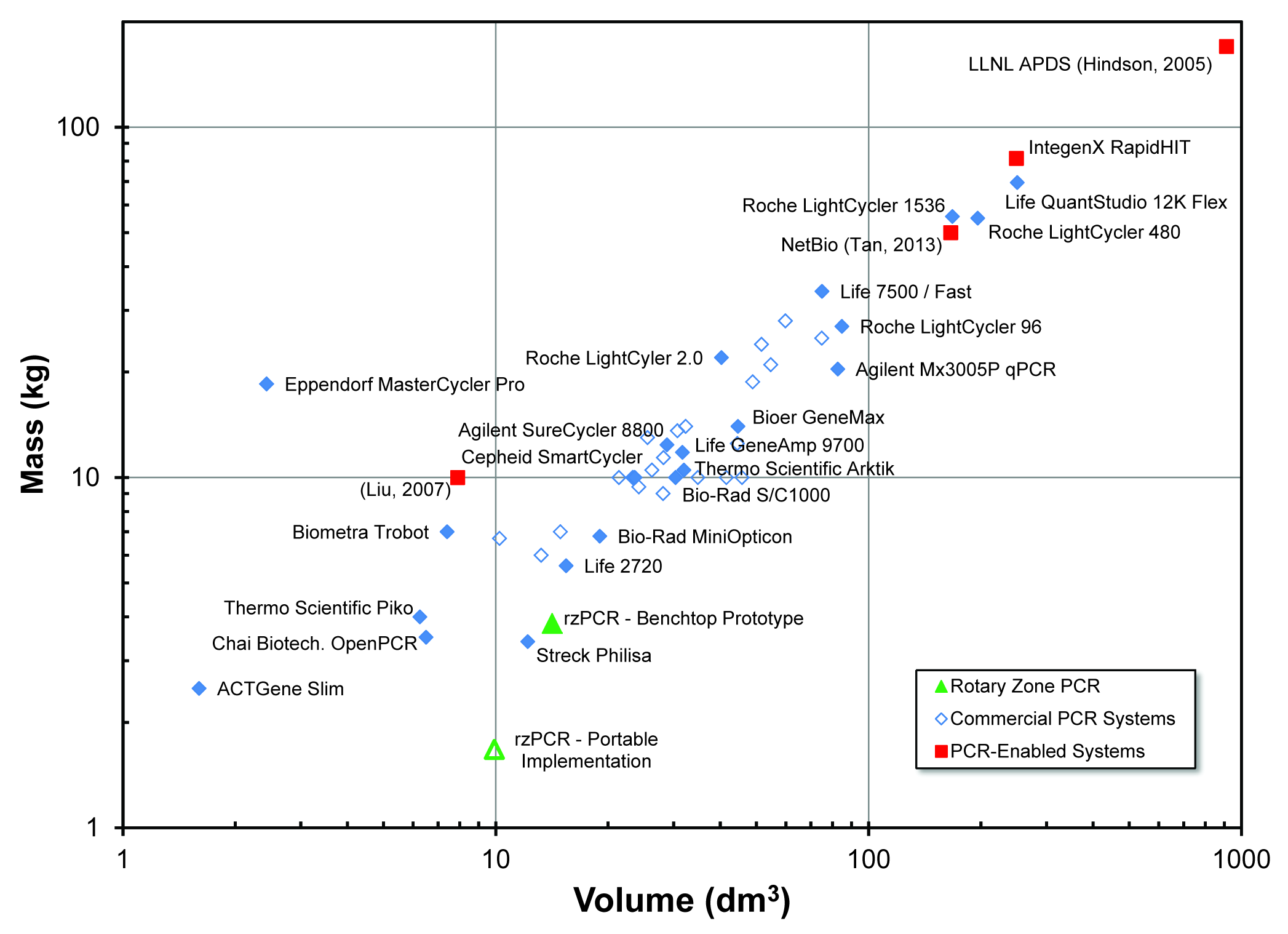

Supplement: S1 Fig — (TIF) [file pone.0118182.s001.tif]

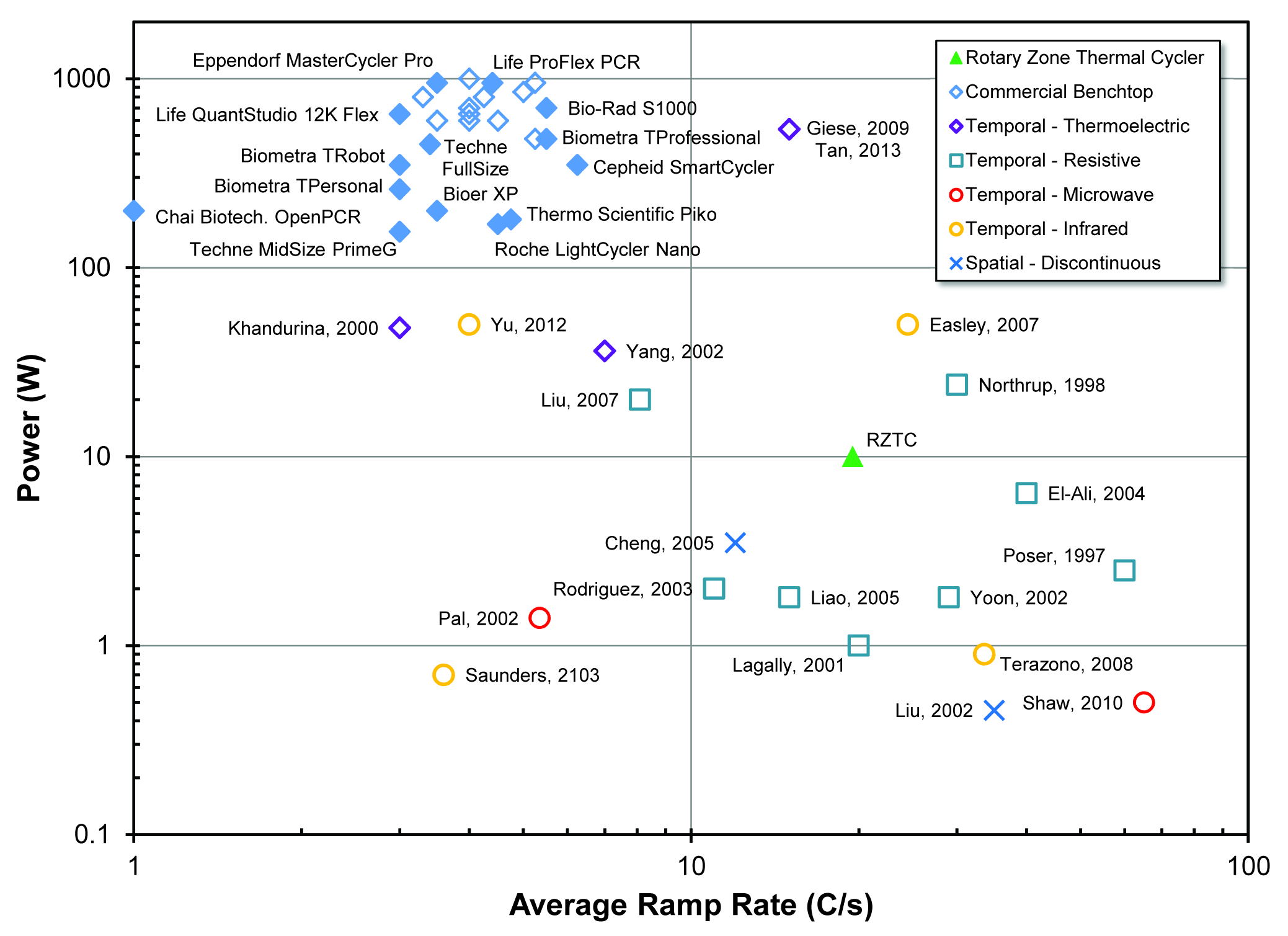

Supplement: S2 Fig — (TIF) [file pone.0118182.s002.tif]

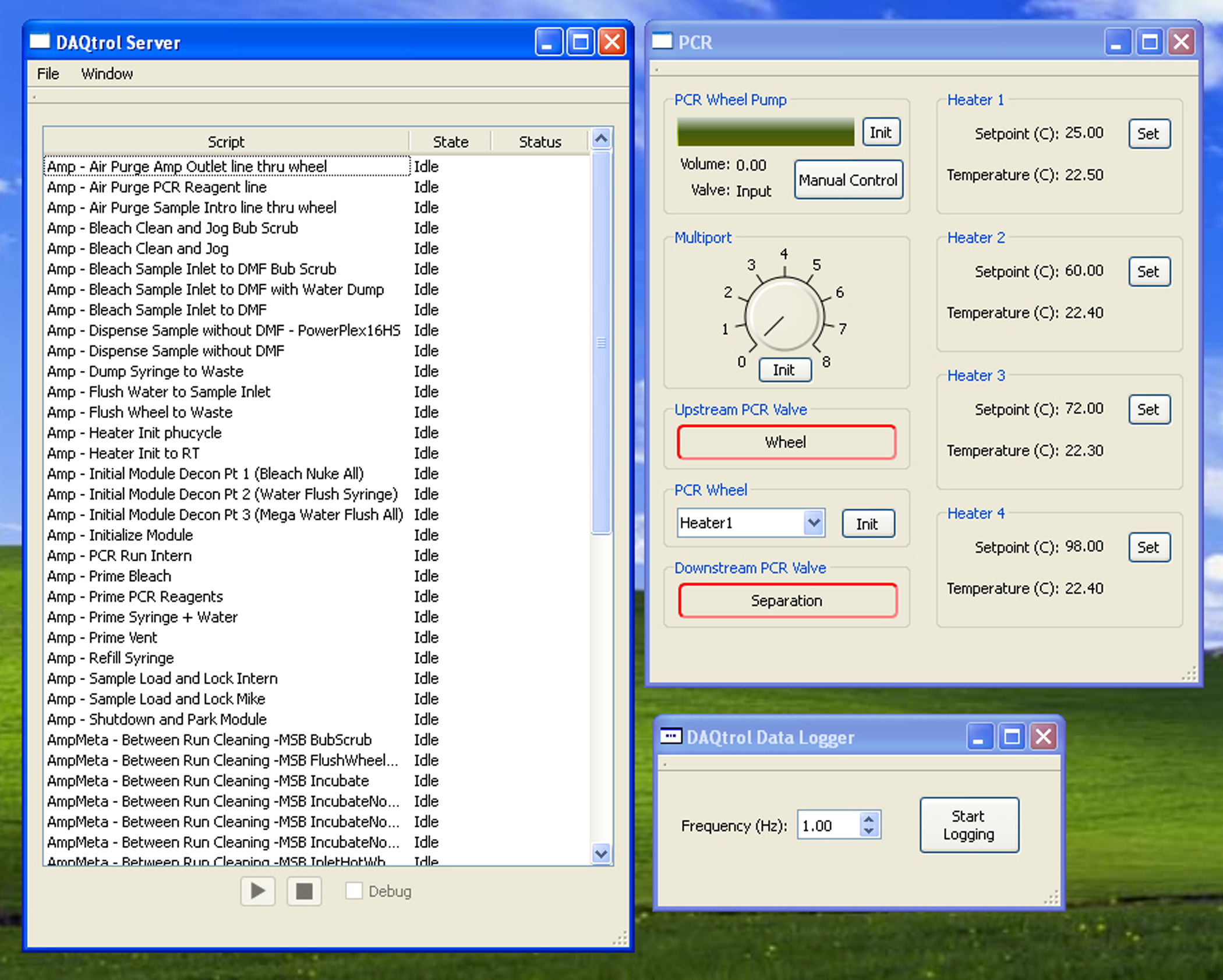

Supplement: S3 Fig — Automated, script-based commands are selected and executed in the window at left, while manual control over system components and temperature data acquisition are provided by the windows at right. (TIF) [file pone.0118182.s003.tif]

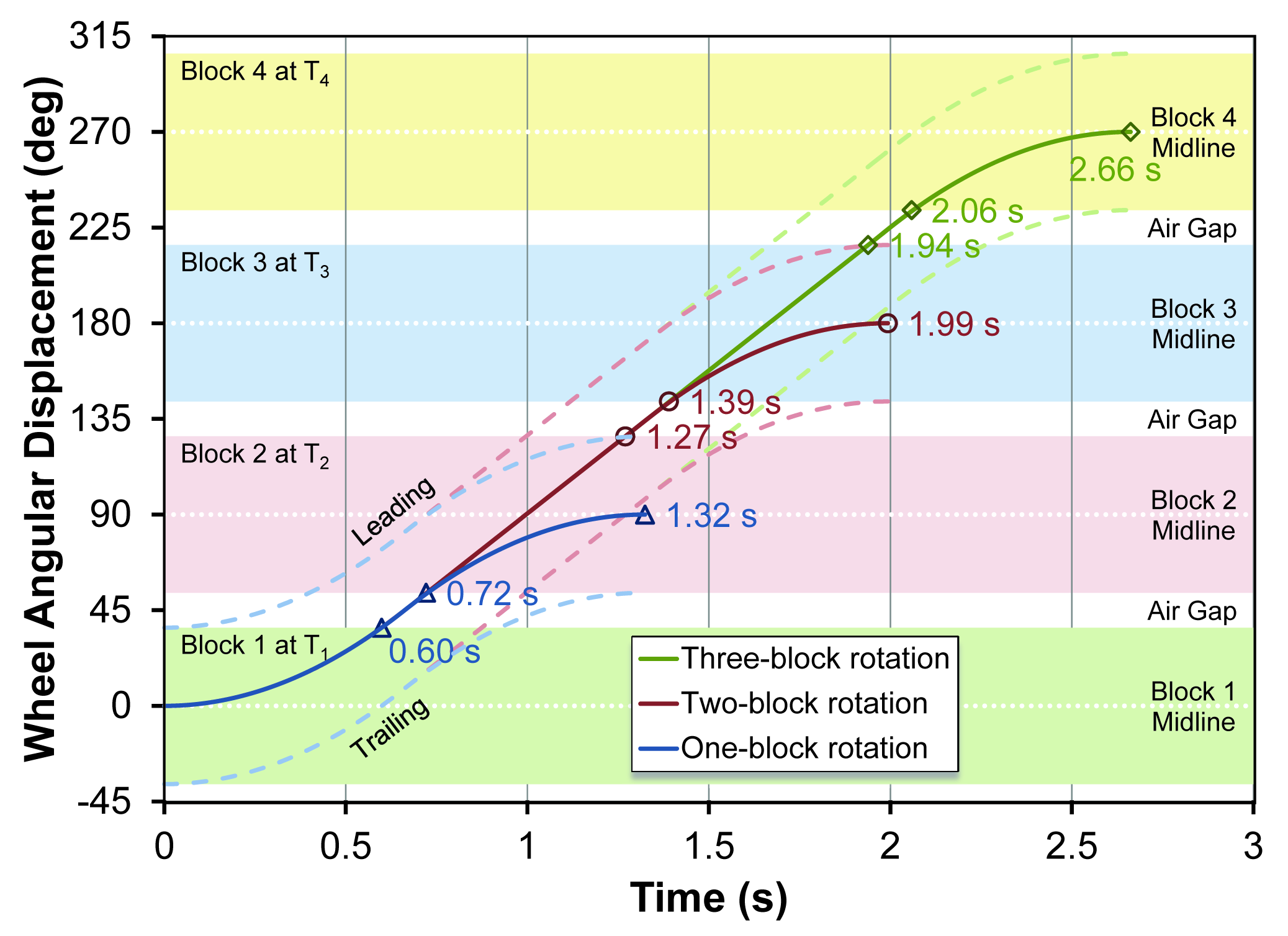

Supplement: S4 Fig — Solid lines show the progression of the underlying heater blocks relative to a point at the center of the fixed sample bolus as a function of time for one-, two-, and three-block transitions. Dashed lines show the temperature history experienced concurrently by points at the leading and trailing end of the same sample bolus. (TIF) [file pone.0118182.s004.tif]
