# Supplementary material for: The Rotary Zone Thermal Cycler: A Low-Power System Enabling Automated Rapid PCR
Source: PLoS One. 2015 Mar 31;10(3):e0118182. doi: 10.1371/journal.pone.0118182 (PMC4380418; doi:10.1371/journal.pone.0118182)
Supplement: S1 Section — (PDF) [file pone.0118182.s005.pdf]

Section S1: Wheel Rotation and Sample Temperature History

While the mechanical response time of the RZTC wheel is a straightforward function of rotational speed and acceleration, its effect on the temperature history experienced by a given point in the sample bolus during rotation merits some consideration. Supplemental Figure S4 shows calculated and video-confirmed rotational displacement times based on Easy Servo speed and acceleration parameters and graphically referenced to the corresponding angular extent of each RZTC block. As the figure illustrates, one-, two-, and three-block transitions require 1.32, 1.99, and 2.66 s, respectively. Worth noting in the context of the thermocouple measurements described in section 4.1.1 of the main article is that these midline-to-midline transition times do not necessarily reflect how long after the start of actuation a given point within the sample bolus will come into contact with the next temperature block. As an example, for a thermocouple positioned at the midline of the starting block in a single-block transition (as in the experiment), rotation will proceed for 0.60 s before the boundary condition at the thermocouple changes due to the arrival of the air gap between Blocks 1 and 2. The leading edge of block 2 will arrive at the midline thermocouple at 0.72 s, having already passed the leading end of the sample bolus at 0.4 s, but will not reach the trailing end of the sample until shortly before the Block 2 midline stops beneath the thermocouple at 1.32 s. Similarly, the trailing end of the sample bolus (lower dashed line) will spend 70% of the rotation time at the initial Block 1 temperature, while the leading end will spend the same fraction at the final Block 2 temperature. While the nonlinearity of the family of curves in Supplemental Figure S4 can be reduced by increasing the wheel acceleration rate, the variations in temperature history experienced by different portions of the sample bolus during ramping are inherent to the RZTC design. When cycling between only two temperature blocks these variations across the sample bolus will average out in each complete bidirectional thermal cycle. In more typical PCR cycling, however, where a series of three single-block transitions are reversed by a single, faster three-block transition, hysteretic accumulation of temperature history error from one side of the sample bolus to the other could be more significant. In general, these accumulated positional variations will be most relevant in cases where 1) dwell times are not significantly longer than ramp times, 2) samples are especially sensitive to temperature ramping conditions, or 3) cycling is performed on a series of small, discrete sample boluses arrayed along the length of the reaction tube. Analogous temperature and residence time distribution issues have been noted for other micro-PCR approaches [38, 74].
